# Identifying policies and strategies for general practitioner retention in direct patient care in the United Kingdom: a RAND/UCLA appropriateness method panel study

**DOI:** 10.1186/s12875-019-1020-x

**Published:** 2019-09-12

**Authors:** Rupa Chilvers, Suzanne H. Richards, Emily Fletcher, Alex Aylward, Sarah Dean, Chris Salisbury, John Campbell

**Affiliations:** 1 Primary Care Research Group, University of Exeter Medical School, Smeall Building, St Luke’s Campus, Exeter, EX1 2LU UK; 2 Academic Unit of Primary Care, Leeds Institute of Health Sciences, Worsley Building, Clarendon Way, Leeds, LS2 9NL UK; 3 University of Exeter Medical School, College House, St Luke’s Campus, Exeter, EX1 2LU UK; 4 Centre for Academic Primary Care, Population Health Sciences, University of Bristol, Bristol, BS8 2PS UK

**Keywords:** Primary care physicians, Health workforce, Work engagement, Job description, Staff development, Personnel turnover, Health care reform, Consensus method

## Abstract

**Background:**

The United Kingdom (UK) is experiencing a general practitioner (GP) workforce retention crisis. Research has focused on investigating why GPs intend to quit, but less is known about the acceptability and effectiveness of policies and strategies to improve GP retention. Using evidence from research and key stakeholder organisations, we generated a set of potential policies and strategies aimed at maximising GP retention and tested their appropriateness for implementation by systematically consulting with GPs.

**Methods:**

28 GP Partners and GPs working in national stakeholder organisations from South West England and London were purposively sampled, and asked to take part in a RAND/UCLA Appropriateness Method panel. Panellists were asked to read an evidence briefing summary, and then complete an online survey on two occasions. During each round, participants rated the appropriateness of policies and strategies aimed at improving GP retention using a nine point scale (1 ‘extremely inappropriate’ to 9 ‘extremely appropriate’). Fifty-four potential policies and strategies (equating to 100 statements) were tested, focusing on factors influencing job satisfaction (e.g. well-being, workload, incentives and remuneration, flexible working, human resources systems). Ratings were analysed for panel consensus and categorised based on appropriateness (‘appropriate’, ‘uncertain’, ‘inappropriate’).

**Results:**

12/28 GPs approached agreed to take part, 9/28 completed two rounds of the online survey between February and June 2018. Panellists identified 24/54 policy and strategy areas (41/100 statements) as ‘appropriate’. Examples included providing GP practices ‘at risk’ of experiencing GP shortages with a toolkit for managing recruitment and retention, and interventions to facilitate peer support to enhance health and wellbeing, or support portfolio careers. Strategies to limit GP workload, and manage patient demand were also endorsed.

**Conclusions:**

The panel of experienced GPs identified a number of practical ways to improve GP retention through interventions that might enhance job satisfaction and work-life balance. Future research should evaluate the impact of implementing these recommendations.

**Electronic supplementary material:**

The online version of this article (10.1186/s12875-019-1020-x) contains supplementary material, which is available to authorized users.

## Background

Demand for UK general practice-based primary care has consistently risen over the last 7 years, with the largest increase (13.6%) observed in consultation rates for general practitioners (GPs) [1]. The UK is also experiencing a shortfall in the GP workforce, as evidenced by a substantial increase in the number of unfilled GP full-time posts (from 2.1 to 7.9% between 2010 and 2013) [2], and a shift toward part-time working within the existing workforce [3]. Numerous workforce surveys have highlighted a retention crisis; a conservative estimate is that in the next 5 years around a third of GPs intend to quit, reduce hours or seek alternatives to working in direct patient care [4, 5]. International evidence has identified that a strong primary care-based healthcare system is associated with improved patient satisfaction with care, reductions in population health inequalities and adverse health outcomes, and reduced health care costs [6]. Given that 90 % of NHS patient contact takes place in general practice, and almost two thirds of contacts are with GPs, there is an urgent need to maximise UK GP retention to protect the quality of health care provided.

A systematic review of qualitative and quantitative evidence was undertaken as part of the ReGROUP project in order to summarise the factors related to GPs quitting and/or intending to quit patient care [7]. This review identified a number of ‘push’ and ‘pull’ factors which influenced decisions to reduce hours or leave direct patient care. Job dissatisfaction and work-related stress (e.g. lack of autonomy, rising patient demand) were ‘push factors’ discouraging continuation in direct patient care. The desire to pursue other interests outside of work as well as a culturally acceptable norm to retire early were pull factors towards quitting or reduced hours. Negative perceptions about being a GP within the current environment of UK general practice also appeared to discourage GPs from returning to direct patient care following a career break. The review findings were used to inform the design of potential recruitment and retention interventions specifically aiming to improving job satisfaction and addressing issues relating to stress in the work place.

The evidence regarding the effectiveness of interventions aimed at improving GP retention that is directly applicable to the UK setting is sparse [8]. Barriball et al. (2015) [9] synthesised the international literature on recruitment and retention practices for healthcare professionals. The interventions were classified using the WHO (2010, [10] categories relating to: education; contracts and regulation; financial incentives; or professional and personal support. Around a third (*n* = 39) of recruitment and retention interventions were tested in the UK, and some were multifactorial, combing two or more categories. The authors concluded that based on the evidence available, single interventions appeared to have limited effects on GP retention over time [9]. Similar reviews have reported that higher wages appear to have an initial positive influence on job satisfaction, the effectiveness of financial incentives on retention declines after 5 years [11, 12]. In a review of reviews, Misfeldt et al. (2014) concluded that improving the work environment and instituting mechanisms for work-life balance improved human resource outcomes in addition to the use of financial incentives [12].

The limited evidence base for effective interventions for retention was further illustrated in a recent systematic review of the strategies to recruit and retain doctors in primary care [13]. The authors identified 51 studies (42 interventions), mainly derived from USA, Canada and Australia, presenting strategies under the broad categories of: retainer schemes; re-entry schemes; support for professional development or research; specialised recruiters or case managers; well-being or peer support initiatives; and financial incentives. All studies were judged to be of low methodological quality, precluding any definitive conclusions regarding the effectiveness of such interventions.

The present study was part of the mixed methods ReGROUP project [7], with earlier work streams including a systematic literature review and qualitative interview study [14]. Here we report the findings of a panel consensus study, the aim of which was to identify policies and strategies that might be potentially appropriate at facilitating the retention of GPs in direct patient care in the UK.

## Methods

We sought to identify emergent policies and strategies supporting the retention of GPs in direct patient care, considered from the perspective of GP Partners (e.g. GPs responsible for leadership and management within their practice). Expert consensus methods, such as modified Delphi techniques, have been used for the development of clinical guidelines [15, 16], to inform UK policy and organisational interventions [17, 18], and to rank strategies for recruitment and retention of rehabilitation professionals in Ontario, Canada [19]. We adopted the RAND/UCLA Appropriateness Method (RAM) [20] in which expert panel members use their professional judgement alongside the best available evidence to identify areas where consensus can be reached for the topic under consideration.

### Sampling considerations

The panel comprised GPs directly responsible for managing GP recruitment/retention including GP Partners and GPs working in a national role in workforce planning. Consistent with RAM methodology, which works in-depth with a small number of participants, we aimed to recruit between seven and 15 GPs to take part as panel members. Potential participants were sampled from a high population density area (London), and urban and rural areas (South West England) in the UK. Purposive sampling was used to identify approximately 40 partners who were eligible to take part in the study. GPs who had contributed to other work streams within the ReGROUP project were excluded to avoid individuals being invited to participate in the same research project multiple times.

Eligible participants were identified in South West England using the ‘Medical Performers List’ of all GPs registered to practise in this area as of March 2016 (3523 GPs). A randomly generated list of 34 partners was sampled with equal numbers identified from urban and rural settings. As the equivalent list was not available to the researchers for the high population density areas in London, a database was compiled using publicly available information. The 12 Clinical Commissioning Groups (CCGs) with the highest population density in London were identified, and within each CCG area a list of practices was compiled (by list size). The sample of 16 practices on the list were then selected at random and the names of individual GP Partners were extracted from the websites. In each practice, one partner was selected by the researcher (RC) to be contacted (*n* = 16). The resultant list of 50 potential participants was randomised, with the first 25 names (16 from the South West, 9 from London) being invited to participate. The remaining individuals were retained to supplement sampling if the recruitment proved challenging.

National GP representatives were identified through a snowballing technique. Policy or strategy leads from key stakeholder organisations working across England were approached, including: the Regional Offices of Health Education England (HEE) having oversight of postgraduate GP training; the British Medical Association (BMA), Royal College of General Practice (RCGP), and the Nuffield Trust. Three potential participants were identified through this process and were invited to take part in the study.

### Recruitment of panel members

Selected potential participants were sent a recruitment pack including a covering letter and participant information sheet; where possible, invitations were sent electronically rather than by post. An online link was provided for participants to confirm their willingness to be contacted. A reminder was sent within 2 weeks to non-respondents. Following agreement to participate, members were provided further information via email, and informed of the dates of the two rounds of data collection.

### Developing the survey

Three sources of information were used to develop policies and strategies for panel consideration: (1) research evidence from systematic reviews and key reports; (2) UK policy documents relating to GP recruitment and retention reported by NHS England, the BMA, and the RCGP; and (3) the emergent findings from the ReGROUP systematic review [7] and qualitative studies [14]. The selected papers, reports and policy developments were summarised into a short evidence briefing paper for consideration by the panellists (Additional file 1).

From these evidence sources, combined with the conceptual framework presented in the ReGROUP evidence review [7], we developed a list of inclusion and exclusion criteria (Table 1) to guide the selection and development of policies and strategies considered by panellists.

**Table 1:** Eligibility criteria for the policies and strategies

*Inclusion criteria:*	
1. Policies and strategies extrapolated from key sources regarding areas reported by research, national policy or equivalent publications as relevant to maximising GP retention. The intervention(s) proposed or tested may also be within the context of increasing job satisfaction which was considered to be an influential factor for GP retention. 2. Policies and strategies addressing known barriers and facilitators to increasing GP retention, reducing intention to leave, or encouraging re-entry into direct patient care. 3. Policies and strategies drawn from existing schemes or approaches directed at increasing GP retention, reducing intention to leave, or encouraging re-entry into direct patient care.	
*Exclusion criteria:*	
1. Policies and strategies which did not fit the UK general practice context in terms of how general practice commissioning is managed, and/or GPs and practices provide care. 2. It is known that it would take more than 5 years to implement the relevant policies and strategies (irrespective of whether direct impacts on GP retention rates could be quickly realised there afterwards). 3. Policies and strategies which are not described in current research and policy documents. The latter includes innovations that might be plausibly be used to facilitate GP retention but where were currently untested or not specified within the literature.	

Given that the UK is already experiencing a GP workforce crisis [5], we elected to focus on potential policies and strategies likely to impact on GP retention in the short term, defined here as within 5 years. Preliminary policies and strategies were identified and developed through two facilitated sessions with the ReGROUP project researchers (including academic GPs and work stream leads) and a further session with a group of six patient and public representatives. The policies and strategies were mapped onto 11 topic areas (Table 2), examples of which include health and wellbeing programmes for GPs, encouraging the growth of new GP Practices and systems, and additional support packages specifically for GPs who are reaching retirement age and can take their pension upon exit.

**Table 2:** Summary of the topic areas of policies and strategies presented to the RAM Panel

Implementation level	N policies & strategies	N tested for sub-groups	Sub-groupings^a^	N statements
National/regional level				
1. Supporting areas based on ‘at risk of GP shortages’ status within the next 5 years	10	2	Implementation mode	12
2. Encouraging growth of new GP practices & systems	5	1	Practice setting	6
3. Marketing-based interventions & publicity campaigns	3	0	-	3
GP Practice level				
4. Focussing on GP returners	3	1	Implementation mode	4
5. Flexible working and managed exits	6	0	-	6
6. Human resources management for GPs	5	5	Practice setting	10
GP level				
7. Health and wellbeing	3	3	Pensionable status	9
8. Professional support	3	1, 3	Implementation mode, Pensionable status	8
9. Support for portfolio working	4	1, 4	Implementation mode, Pensionable status	15
10. Employment, contracts and transition	6	6	Pensionable status, GP returners	18
11. Additional support for GPs nearing retirement	6	1	GP role	9
TOTAL	**54**			**100**

^a^Implementation mode = ‘compulsory’ or ‘optional’; practice setting = ‘all practices’ or ‘practices operating in traditionally “hard to recruit” areas; pensionable status = ‘all GPs’, ‘GPs nearing retirement age and who could take their pension’ or ‘GPs not nearing retirement age and could not take their pension’; GP role = ‘GPs who have not encountered any concerns in the previous revalidation or appraisal processes’ or ‘GPs who would like to work with a specified and limited scope of practice’; GP returners = ‘GPs returning to practice’, or ‘newly qualified GPs’

The final set of potential policies and strategies (*n* = 54) (Table 2) were grouped into three broad categories based on whether implementation was expected to take place at a national/regional level (*n* = 18), at the level of GP practice (*n* = 14), or at the level of an individual GP (*n* = 22).

The potential policies and strategies were presented to panellists as short statements. Each statement presented a single intervention or strategy, but for some policies and strategies the panellists were asked to make their ratings for specific sub-groups, which created further statements (Table 2). Possible sub-groups included: implementation mode (2 levels: ‘compulsory’ versus ‘optional’ implementation); practice setting (2 levels: ‘all practices’ or ‘practices operating in traditionally “hard to recruit” areas); pensionable status (3 levels: ‘all GPs’, ‘GPs nearing retirement age and who could take their pension’ or ‘GPs not nearing retirement age and could not take their pension’); GP role (2 levels: ‘GPs who have not encountered any concerns in the previous revalidation or appraisal processes’ or ‘GPs who would like to work with a specified and limited scope of practice’); and GP returners (2 levels: ‘GPs returning to the practice’ or ‘newly qualified GPs’). Half of the 54 policies and strategies presented were applicable to ‘all GPs’ or to ‘all practices’, with the remainder tested in statements applicable to specific sub-groups. Accounting for sub-groups, the panellists were presented with 100 statements to assess. A copy of the questionnaire used, including a list of the statements, is given in the Additional file 2.

For each statement, panellists were asked to rate ‘appropriateness’ using a nine-point scale ranging from 1 (extremely inappropriate) to 9 (extremely appropriate). Participants were advised to rate a statement as ‘appropriate’ when the expected benefits might reasonably be anticipated to exceed the expected risks. The expected benefit was assumed to occur when, after implementation, GPs would be more likely to continue in direct patient care without substantially reducing their working hours. The expected risk in this context was that the potential policy or strategy approach would not impact GPs’ intentions to quit or to substantially reduce their commitment, or that it might result in unintended consequences that might exacerbate the retention problem. When rating for appropriateness, participants were specifically instructed not to consider the cost implications; the consideration of benefits and risks should consider issues relating to access, equity, and safety of health care, combined with impacts on patient experience.

### Data collection

Panellists were invited to complete two rounds of data collection via an online survey, with paper completion available on request (requested by one panellist). The first round took place in February 2017, and the second in April 2017.

One week before round one, participants were emailed completion instructions, with a unique username and password, and with an electronic copy of the supporting evidence summary. The online survey for round one included the 54 potential policy and strategy areas presented as 100 statements for rating. Participants were asked to read the evidence summary and then use their professional judgement to rate the ‘appropriateness’ of each statement. All participants had the contact details for the research team, who they could contact if they had any uncertainties regarding the materials provided. Participants had four weeks to complete the survey, with a reminder email sent to non-responders within ten days. An interim descriptive analysis was then undertaken to allow data to be fed back to panellists during round two of data collection.

In round two, each participant was sent an online link for the second online survey. Participants were shown the whole group’s ratings as frequency data on a rating scale for each item, alongside their own ratings for each statement from round one. Consistent with the RAM process, participants could revise their own original rating in light of the group ratings if they so wished. Participants had three weeks to complete round two, with a reminder sent to non-responders after ten days and three weeks.

### Data analysis

In line with the RAM method [20], the panel median score was calculated for each statement and classified into three bands: 1–3.5 (potentially inappropriate); 3.6–6.4 (uncertain), and 6.5–9 (potentially appropriate). Using this more inclusive approach, a narrower median band was applied for the ‘uncertain’ classification (i.e. more statement were deemed appropriate/inappropriate as opposed to uncertain) as the statements related to informing policy decisions rather than to informing clinical decision-making which might directly impact on patient safety and harms.

For interpretation, the degree of consensus between panellists was also taken into account. Consensus was judged to be achieved when no more than two panellists provided ratings for a statement outside of the band in which the group median score was located. For example, for a statement to be deemed ‘appropriate’, the panel median score must fall between 6.5 and 9 with consensus, i.e. no more than two panellists giving ratings of between 1 and 6.4.

If the panel did not reach consensus then the statement was interpreted to be of uncertain or equivocal value, despite the panel median score. Although analysis took place for individual statements (*n* = 100), results are presented at the level of the 54 policy and strategy areas.

## Results

Twelve of the 38 GPs approached (31%) agreed to take part, ten of whom (26%) completed round one (two did not respond after reminder emails). Participants included five GP Partners from the South West, three from London and two from national organisations. Nine of the ten participant GPs completed round two after reminders emails (9/38,24%).

### Panel ratings

**Fig. 1:**
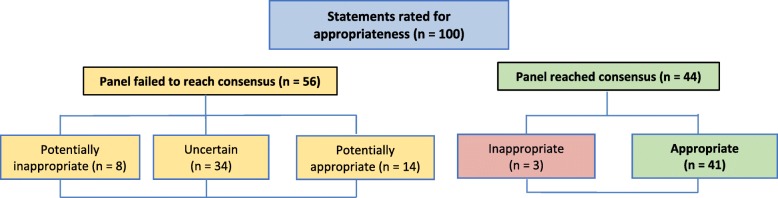
The data collection process for ratings for appropriateness of the 100 statements after two rounds of voting

A summary of the panellist responses to statements after the two rounds of data collection is presented in Fig. 1, with specific responses to individual statements summarised in Tables 3 and 4.

**Table 3:** Panellist median scores for policies and strategies deemed ‘appropriate’ or ‘inappropriate’ after accounting for panel consensus

ID	Policy and strategy assessed by panellists	Median ^a^
	*For implementation at national/ regional level (n = 6)*	
1	In order to assess ‘at-risk of GP shortages’ status in a commissioning/planning area and taking into account confidentiality GP practices should be able to self-register their organisation’s ‘at-risk’ status.	8
2	GP practices identified as being ‘at-risk’ of GP shortages should be provided with a toolkit to manage recruitment and retention.	8.5
3	New incentive and support packages should be available to GPs and other organisations setting up new practices or new ways of working in under-doctored areas.	7.5
4	There should be a publicity campaign focussing on managing expectations of patients in line with the resources and constraints of GP-based primary care services.	9
5	GP practices identified as being ‘at-risk of GP shortages’ should be managed with an appropriate and sensitive supportive arrangement *– for (i) optional implementation.*	8 ^b^
6	GP practices identified as being ‘at-risk of GP shortages’ should be allocated a specialist team for managing recruitment and retention *– for (i) optional implementation.*	9 ^b^
	*For implementation at GP practice level (n = 4)*	
7	GPs who are returning to work after a period of absence or after a career break should have access to ‘Health and Wellbeing programmes’ to help them manage their re-entry into the workforce – *for (i) optional implementation.*	8.5 ^b^
8	GPs who are returning to work after a period of absence or after a career break should have access to schemes that have a range of routes and options that can be combined in a personal package for re-entry.	9
9	GPs who are returning to work after a period of absence or after a career break should have access to schemes that use a mix of online education and face-to-face meetings to ensure timely access to induction and refresher courses.	9
10	GP practices should implement strategically planned exits for retiring GPs.	7
	*For implementation at GP level (n = 14)*	
11	Peer support initiatives should be made available to GPs aimed specifically at health and well-being *- for (i) GPs who are not reaching retirement age.*	8.5 ^b^
12	GPs should have access to their own specialised health care service to ensure a quick and confidential occupational healthcare service *– for (i) all GPs, (ii) GPs reaching retirement and who could take their pensions, or (iii) GPs not reaching retirement.*	9, 9, 9 ^b^
13	A structured programme of training and support should be made available to all GPs in their first 5 years following qualification as an independent GP to help them establish healthy, productive careers *– for (i) optional implementation.*	7 ^b^
14	GPs should consider portfolio working as part of their career pathway and this should be optional *- for (i) all GPs, (ii) GPs reaching retirement and who could take their pensions, or (iii) GPs not reaching retirement.*	9, 7, 7 ^b^
14	GPs should consider portfolio working as part of their career pathway and this should be compulsory *- for (i) all GPs, (ii) GPs reaching retirement and who could take their pensions, or (iii) GPs not reaching retirement.*	1, 1, 1 ^b^
15	Career support should be available to GPs to enable portfolio opportunities to be identified and taken up in a strategic way to inform their future ambitions - *for (i) all GPs, or (ii) GPs not reaching retirement.*	8, 7.5 ^b^
16	Incentives and support packages should be available for those GPs developing portfolio careers who are linking their portfolio activities to specialisms/areas that are directly beneficial to local clinical priorities - *for (i) all GPs, (ii) GPs reaching retirement and who could take their pensions, or (iii) GPs not reaching retirement.*	8, 8.5, 8.5 ^b^
17	Where a strong case can be made that there is a financial risk directly relating to the work of the practice (e.g. ownership of premises), GPs should have access to schemes to reduce financial burden (e.g. buy back schemes for premises) *– for (i) all GPs or (ii) GPs reaching retirement and who could take their pensions*	9, 9 ^b^
18	There should be an agreed maximum in the number of consultations that a GP should be allowed to conduct in a working day in order to protect patient safety as well as the health of the GP - *for (i) all GPs, (ii) GPs reaching retirement and who could take their pensions, or (iii) GPs not reaching retirement.*	9, 9, 9 ^b^
19	There should be contractual changes to encourage longer consultations where appropriate - *for (i) all GPs, (ii) GPs reaching retirement and who could take their pensions, or (iii) GPs not reaching retirement.*	9, 9, 9 ^b^
20	The working hours of GPs should routinely include fully-funded, dedicated time to accommodate the full range of roles (administrative, clinical, training, management, CPD, business undertaken as part of care professional activity *– for (i) all GPs or (ii) GPs reaching retirement and who could take their pensions.*	9, 9, 9 ^b^
21	Contracts based on specified programmed activities should be available to GPs to work across several GP practices and on other health related activities *– for (i) all GPs, (ii) GPs reaching retirement and who could take their pensions, or (iii) GPs not reaching retirement.*	7, 8, 8 ^b^
	*Specifically regarding GPs who are reaching retirement and who could take their pensions on exit*	
22	For such GPs a comprehensive flexible careers scheme should be introduced with a view to supporting annualised hours, part-time working, and/or ad-hoc contributions to direct patient care.	9
23	For such GPs there should be financial incentives for such GPs who have maintained a prolonged/sustained period of direct patient care.	8.5
24	The annual appraisal and revalidation process should be reviewed with a view to streamlining and simplifying the process *- for (i) GPs who have not encountered any concerns in the previous revalidation/appraisal processes, or (ii) GPs who would like to work with a specified and limited scope of practice.*	8.5, 8.5 ^b^

^a^The median scores are presented for the statements where the panellists reached consensus i.e. ≤ 2 panellists’ ratings were outside the ‘appropriate’ range band (7–9) or ‘inappropriate’ range band (1–3)

^b^The median scores presented are for the sub-groups presented in italics at the end of each policy and strategy area deemed to be ‘appropriate’ or ‘inappropriate’; where applicable, the other levels of the sub-group deemed ‘uncertain’ by panellists are presented in Table 3

**Table 4:** Policies and strategies deemed of ‘uncertain’ value after accounting for panel consensus

ID	Policy and strategy assessed by panellists	Median
	*For implementation at national/ regional level (n = 14)*	
25	In order to assess ‘at-risk’ status in a commissioning/planning area and taking into account confidentiality, GPs should be required to provide ‘intention to quit’ information regularly to assess areas ‘at-risk’.	3
26	In order to assess ‘at-risk’ status in a commissioning/planning area and taking into account confidentiality, GPs should be required to complete job satisfaction surveys (or equivalents) regularly to assess areas ‘at-risk’.	4.5
27	In order to assess ‘at-risk’ status in a commissioning/planning area and taking into account confidentiality, GP practices should be required to register their organisation’s at-risk status.	5
28	In order to assess ‘at-risk’ status in a commissioning/planning area and taking into account confidentiality: there should be regular audits to identify GP practices ‘at-risk’.	8 ^a^
29	GP practices identified as being ‘at-risk’ should be targeted with additional support and incentives.	7.5 ^b^
30	GP practices identified as being ‘at-risk’ should be prioritised for new/innovative national schemes to support GP retention and/or return to work.	7 ^b^
5	GP practices identified as being ‘at-risk’ should be managed with an appropriate and sensitive supportive arrangement *– for (i) compulsory implementation.*	3 ^a b^
6	GP practices identified as being ‘at-risk’ should be allocated a specialist team for managing recruitment and retention *– for (i) compulsory implementation.*	4.5 ^a^
31	New arrangements should be developed so that GPs can become more involved in GP practice management without being partners.	5.5
32	New business models should be developed for GPs who wish to provide care within the NHS but prefer not to own a GP practice.	5
33	There should be incentive and support packages for not-for-profit organisations employing GPs to work across GP practices.	5
34	Hospitals should be permitted to open GP practices with registered lists – *for (i) all areas, or (ii) operating in traditionally “hard to recruit” settings.*	4, 5.5 ^b^
35	There should be a publicity campaign highlighting the experiences of GPs who have successfully been retained in direct patient care as part of a marketing-based intervention aimed at GPs.	4.5
36	The positive experiences of GPs who are providing direct patient care should be consistently shared in a number of ways such as blogs and articles as part of a marketing-based intervention aimed at GPs.	5
	*For implementation at GP practice level (n = 12)*	
7	GPs who are returning to work after a period of absence or after a career break should have access to ‘Health and Wellbeing programmes’ to help them manage their re-entry into the workforce – *for (i) compulsory implementation.*	4.5 ^a^
11	Peer support initiatives should be made available to GPs aimed specifically at health and well-being *- for (i) all GPs or (ii) GPs reaching retirement and who could take their pensions.*	9, 8.5 ^a b^
37	GP practices should have systems in place to accommodate flexible ways of working.	7 ^b^
38	GP practices should be able to demonstrate commitment to flexible ways of working through written human resources policies, guidelines or equivalents.	5
39	Human resources management support should be available to GP practices who are actively supporting GPs in combining other career interests with direct patient care.	7 ^b^
40	GP practices should receive guidance on recommended approaches to supporting the staged exit of GPs who are looking to leave direct patient care.	7 ^b^
41	GP practices should receive a toolkit on recommended approaches to supporting the staged exit of GPs who are looking to leave direct patient care.	5.5
42	Human resources responsibilities should be carried out externally to the employer/practice with responsibility for ongoing monitoring of how many GPs within an area have requested and successfully implemented flexible working arrangements – *for (i) all GP practices, or (ii) GP practices operating in traditionally “hard to recruit” settings.*	2.5, 5 ^a b^
43	Human resources responsibilities should be carried out externally to the employer/practice with responsibility for managing flexible working arrangements for GPs – *for (i) all GP practices, or (ii) GP practices operating in traditionally “hard to recruit” settings.*	2.5, 5 ^a b^
44	Human resources responsibilities should be carried out externally to the employer/practice with responsibility for all activities associated with retention of GPs – *for (i) all GP practices, or (ii) GP practices operating in traditionally “hard to recruit” settings.*	3, 5 ^a b^
45	Human resources responsibilities should be carried out externally to the employer/practice with responsibility for all activities associated with professional development and training – *for (i) all GP practices, or (ii) GP practices operating in traditionally “hard to recruit” settings.*	2, 3 ^a b^
46	Human resources responsibilities should be carried out externally to the employer/practice with responsibility for implementing standards for working hours and conditions – *for (i) all GP practices, or (ii) GP practices operating in traditionally “hard to recruit” settings.*	5, 5 ^a^
	*For implementation at GP level (n = 12)*	
47	GPs should have access to their own specialised health care service to ensure a quick and confidential general health service – *for (i) all GPs, (ii) GPs reaching retirement and who could take their pensions, or (iii) GPs not reaching retirement.*	5.5, 5.5, 5.5 ^a^
13	A structured programme of training and support should be made available to all GPs in their first 5 years following qualification as an independent GP to help them establish healthy, productive careers *– for (i) compulsory implementation.*	3 ^a^
48	GPs should receive business management training and opportunities as a component of updating their skillsets - *for (i) all GPs, (ii) GPs reaching retirement and who could take their pensions, or (iii) GPs not reaching retirement.*	6, 5, 6 ^a^
49	Clinical mentorship should be available to GPs as part of a nationally managed scheme - *for (i) all GPs, (ii) GPs reaching retirement and who could take their pensions, or (iii) GPs not reaching retirement.*	6.5, 6, 6 ^a^
15	Career support should be available to GPs to enable portfolio opportunities to be identified and taken up in a strategic way to inform their future ambitions *– for (i) GPs reaching retirement age and could take pensions*	7 ^a b^
50	Incentives and support packages should be available for those GPs developing portfolio careers who are making a substantial contribution to direct patient care service - *for (i) all GPs, (ii) GPs reaching retirement and who could take their pensions, or (iii) GPs not reaching retirement.*	7, 8, 7 ^a b^
17	Where a strong case can be made that there is a financial risk directly relating to the work of the practice (e.g. ownership of premises), GPs should have access to schemes to reduce financial burden (e.g. buy back schemes for premises) *– for (i) GPs not reaching retirement*.	7 ^a b^
51	GPs should be expected to include regular supervision/mentoring sessions as part of their normal professional activity - *for (i) all GPs, (ii) GPs reaching retirement and who could take their pensions, or (iii) GPs not reaching retirement.*	6, 5.5, 6 ^a^
	*Specifically regarding GPs who are reaching retirement and who could take their pensions on exit*	
24	The annual appraisal and revalidation process for such GPs should be reviewed with a view to streamlining and simplifying the process – *for (i) all GPs*	5 ^a^
52	Such GPs should be eligible for and offered support to facilitate direct patient care including additional dedicated administrative support.	6
53	Such GPs should be eligible for and offered support to facilitate direct patient care including medical assistants and other equivalent roles.	7 ^a b^
54	Planned exits for such GPs should include pairing them in job share scheme with – (*i) GPs returning to practice, or (ii) newly qualified GPs.*	5, 6 ^a^

^a^The median panel scores are presented are for the sub-groups presented in italics at the end of each policy and strategy area

^b^It is possible for a median score to fall within the ‘appropriate’ range (7–9) or ‘inappropriate’ range (1–3), but the statement to be of uncertain value as the panel failed to reach consensus (i.e. > 2 panellists provided a rating within the required range)

When analysing at the level of policies and strategies, 24/54 areas (equating to 41 statements) were deemed appropriate after round two for at least one of the statements tested (Table 3, IDs 1–24). Fourteen of the 24 policy and strategy areas classified as appropriate were aimed at the level of the individual GP, with a focus on providing additional support and incentives to remain in direct patient care. Of the remainder, four areas were deemed suitable of implementation at the level of the GP Practice and six at the national or regional level. One policy and strategy area (comprising three statements) was deemed appropriate for optional implementation but was rejected if compulsorily implemented; portfolio working as part of a GP’s career pathway was deemed inappropriate if compulsorily implemented, regardless of the career stage of the GP (Table 3, ID = 14).

For eight of the 24 policy and strategy areas deemed ‘appropriate’ in at least one supporting statement, there were also statements deemed of ‘uncertain’ value for at least one sub-group level (Table 3, IDs = 5, 6, 7, 11, 13, 15, 17, 24). Twenty areas were deemed to be of ‘uncertain’ value for all the sub-groups/statements tested (Table 4, IDs = 25–54), including twelve for national/regional implementation, ten for GP practice level implementation and eight for GP-level implementations.

### Impact of sub-groups on panel assessments of appropriateness

Differences emerged based on whether policies and strategies were presented as optional or compulsory modes of implementation. Five areas (IDs: 1, 2, 7, 13, 14) included statements where implementation was presented as being either ‘compulsory’ or ‘optional’. Statements were rated as uncertain for appropriateness (ID: 1, 2, 7, 13) or inappropriate (ID 14) when the policies and strategies were presented as compulsory, but rated as appropriate when presented as optional.

Panellists ratings of statements within policy and strategy areas for other sub-groups tested found the appropriateness ratings were not influenced by sub-groups (GP practice settings, GP roles, or GP returner status), and either were deemed universally appropriate (ID 24), or of uncertain value (ID 34, 42, 43–46, 54).

With regard to the sub-group of a GP’s pensionable status, appropriateness ratings of policy and strategy areas were also broadly consistent i.e. deemed appropriate (ID 12, 14, 16, 18–21) or of uncertain value (ID 47–51). However there were three areas where the pensionable status of the GP yielded different panellist ratings (ID 11, 15, 17). Peer support initiatives for GPs aimed specifically at supporting health and wellbeing (ID 11) were deemed as appropriate for GPs ‘not nearing retirement age’, but panellists were uncertain regarding the appropriateness of such statements for GPs who are ‘reaching retirement age’ or for ‘all GPs’ regardless of the stage of their career. Similarly, career support for GPs wishing to take up portfolio working (ID 15) was deemed appropriate for ‘all GPs’ and ‘those not nearing retirement age’, but was judged to be of uncertain value for GPs ‘reaching retirement age’. Finally, GPs being given access to schemes to reduce financial burden where a strong case can be made that there is a financial risk directly relating to the work of the practice (ID 17) was deemed appropriate for ‘all GPs’ and for ‘GP reaching retirement age’, but of uncertain value for GPs ‘not nearing retirement age’.

## Discussion

Using a consensus method, we identified policies and strategies that might be quickly implemented in the UK to support the retention of GPs in direct patient care, and hence have the potential to ameliorate the current GP workforce crisis within the next five years. The panellists were GPs responsible for workforce planning, either within their own practices, or through national organisations with a vested interest in identifying solutions to this problem.

### Main findings

The panel deemed at least one statement as appropriate for 24 out of 54 policy and strategy areas (see Tables 3 and 4 for a list of statements supported), most of which related to the provision of personal or professional support for GPs to potentially protect against burn-out and improve job satisfaction [8, 9]. Examples include policies and strategies to enable flexible working (e.g. portfolio careers, or support for ‘programmed health care activities’ to allow GPs to work across several GP practices), access to a dedicated occupational healthcare services, and/or peer support schemes. Policies and strategies considered as appropriate for implementation at national level included support for practices identified as being ‘at-risk’ of GP workforce shortages within five years (e.g. a toolkit to support recruitment and retention). Practice-level interventions judged to be appropriate included supporting GPs who are returning to work following a career break, and the need to develop mechanisms for strategically planned exits for retiring GPs. Finally, consistent with improving work-life balance, the panel supported interventions aimed at tackling high GP workloads (e.g. recommending a maximum number of consultations per GP per day, and/or offering longer consultation times per patient), although these areas may prove difficult to implement within the current contractual model. National/regional publicity campaigns to manage patient expectations of primary care capacity were endorsed as a means of managing demand for primary care GP services.

The panel were asked to rate the appropriateness of some of the policy and strategy areas when applied to specific sub-groups of GPs or GP practice settings. By and large, the panel did not appear to differentiate between sub-groups, consistently providing ‘appropriate’ or uncertain’ ratings across all related statements. However, there were two notable exceptions. First, the panel deemed ‘optional’ implementation to be appropriate for five policy and strategy areas, but ‘compulsory’ implementation was not supported, suggesting that compulsory implementation of interventions is likely to meet with resistance from GPs. Second, as the GP workforce is ageing and the numbers eligible for early retirement are rising [2], we evaluated interventions to incentivise GPs to remain in direct patient care rather than take early retirement. Although we found that the panel did not, on the whole, differentiate between GPs sub-groups based on their pensionable status (i.e. targeting all GPs, or sub-groups who may/may not be eligibility to draw a pension), there were exceptions related to peer support for health and wellbeing, and career support through portfolio working interventions. Here the panel deemed it appropriate to target GPs who are unable to draw their pensions as opposed to those approaching retirement with pensions. In contrast, schemes protecting GP practices from financial risk were deemed appropriate for GPs approaching retirement or all GPs, but of uncertain value for GPs who are not of pensionable age.

### Study strengths and limitations

We demonstrated that the RAM approach is a viable method for determining the content for policies and strategies for GP retention, although we acknowledge some important limitations.

Firstly the use of ‘appropriateness’ as the rating scale is not without difficulties in interpretation. We attempted to minimise difficulties in interpretation by providing panelists with carefully worded definitions. While alternative descriptors, such as ‘importance’ [19] and ‘necessity’ [18], could have been used, we believe these terms would pose their own challenges in interpretation. Notwithstanding this, we recognize that the use of alternative terminology for the rating scales may have yielded a different set of potential policies and strategies, or a different understanding or emphasis across the same set of material.

Secondly, the panel was limited to GP Partners from two geographical areas, or GPs involved in national level activities for GP recruitment and retention, and their views may not be representative of other groups of GPs (e.g. salaried doctors, locums) or in different regions of the UK. The strength of our approach to sampling is that participants had a dual role as employers managing the implementation of the selected policies and strategies, as well as being employees and beneficiaries of the support and incentives. Panel consensus is an essential step when identifying ‘appropriate’ strategies using the RAM approach, but there is a trade-off between ensuring the panel is sufficiently homogeneous to maximise consensus, and the degree to which panels represent a broader constituency affected by policies and strategies. Future work in this area might benefit from testing potential policies and strategies with panels composed of primary care commissioners and workforce/resource planners, or salaried and locum GPs or associates.

Thirdly, another potential limitation of this approach was the number of GPs responding to the request to sit on the expert panel; a quarter of those approached completed both rounds of data collection. This may be driven, in part, by the challenges of securing time away from practice to contribute to data collection, and hence our adoption of online survey procedures so that panelists could flexibly fit participation around existing work commitments. While our response rate is comparable to that reported for GPs elsewhere [17], there remains a wider issue of a lack of transparency in methodological reporting, with other authors of consensus studies omitting detail on panel member recruitment [18, 19].

This study took place at a point in time of rapidly changing primary care policy development and innovation in the UK. A strength of the RAM approach was that it allowed the distilling of evidence and the judgements of experienced GPs regarding what might work in the current NHS climate, allowing these views to support stakeholder engagement work held subsequently as part of the ReGROUP project. We sought to identify and assess new policies and strategies that might improve GP retention, as opposed to those that were already being implemented. This was challenging as new announcements were being made regularly, and the detailed content of a given policy or strategy was not readily available. This study took into account all the known developments up until the January 2017 when the panel was convened. It remains possible that there are unintended overlaps between the tested policies and strategies presented here, and that already being adopted in England.

### Implications for research and practice

Over the last two decades, it has been increasingly recognised that addressing healthcare workforce shortages will require a system-wide approach [4, 8]. Despite this, research continues to focus narrowly on four main areas of education, financial, personal and professional support, and regulatory interventions. This RAM study is amongst the first to report on the potential translation of the push and pull factors for GPs quitting direct patient care into wide-ranging potential policies and strategies (which include, but go beyond the four main areas explored previously). The panelists endorsed a number of interventions to facilitate GP retention operating at different points in the healthcare system, such as managing GP workload and contractual requirements, as well as the need for personal and professional support. To reduce response burden, the panel was restricted to assessing policies and strategies to maximise GP retention by targeting areas likely to influence job satisfaction and work-life balance. Thus future research evaluating the appropriateness of other potentially relevant interventions falling outside this scope (e.g. primary care skills mix), and with different GP stakeholders (e.g. salaried GPs, or locums) is warranted.

While this panel of experienced GPs identified potential solutions to ameliorate the GP workforce crisis, the effectiveness of such interventions is often untested and/or supported by only a weak evidence base [8, 9]. The immediacy and magnitude of the current UK GP workforce crisis is such that it is unlikely that new interventions could be robustly tested prior to implementation due to the lengthy timescales required to undertake such evaluations. However, it remains important that the impact of new policies and strategies are evaluated using efficient study designs (e.g. use of routine data and carefully selected performance indicators), and that investigators and policy makers remain alert to potential for both intended and unintended consequences of interventions aimed at maximising GP retention.

## Conclusions

This study identified 54 policies and strategies that might facilitate retention of GPs in direct patient care, 24 of which were deemed appropriate by a panel of GP Partners. These policies and strategies targeted different areas within the complex system of English primary care that might enhance job satisfaction and work-life balance, areas which may be taken forward for wider stakeholder consultations, and future evaluation research.

## Additional files

Additional file 1: RAM Evidence Briefing paper. (PDF 829 kb)

Additional file 2: RAM questionnaire. (PDF 419 kb)

## Data Availability

Study data are stored in a secure repository at the University of Exeter Medical School. Although not publically available, requests to access the dataset will be considered, and sent to the Chief Investigator Professor John Campbell.

## Abbreviations

BMA: British Medical Association
HEE: Health Education England
NHS: National Health Service
NIHR: National Institute for Health Research
RAM: Rand/UCLA Appropriateness Method
RCGP: Royal College of General Practice
UK: GP General Practitioner
