# Supplementary material for: Identifying policies and strategies for general practitioner retention in direct patient care in the United Kingdom: a RAND/UCLA appropriateness method panel study
Source: BMC Fam Pract. 2019 Sep 12;20:130. doi: 10.1186/s12875-019-1020-x (PMC6743144; doi:10.1186/s12875-019-1020-x)
Supplement: Supplementary file 1 — RAM Evidence Briefing paper. (PDF 829 kb) [file 12875_2019_1020_MOESM1_ESM.pdf]

Assessing the appropriateness and feasibility of policies and strategies for retaining GPs in direct patient care: an evidence summary for RAM Panel Members

Section 1: Introducing the RAM approach

The RAND/UCLA Appropriateness Method (RAM) is an internationally recognised technique using a panel of experts to rate statements for appropriateness (Fitch et al., 2001). The level of agreement between experts is used to inform the content of the policies or practices under development. The RAM panel method is commonly used when the evidence base underlying policies or practices may need to be tailored for a particular context or group before being applied, or when there is limited evidence to support decision-making.

> *"The panel method can synthesize existing knowledge, but it cannot create new knowledge. The results of randomized controlled trials informed the panels' detailed ratings. So did the panellists' own experience and their observation of the experience of others. The panel method is a way to bring together various sources of knowledge in order to assess procedure use." (Brook et al., 1991, p.10)*

In this project, RAM is used to assess the appropriateness and feasibility of policies and strategies for retaining general practitioners (GPs) in direct patient care. This RAM panel is part of a wider ReGROUP project, which was been funded by the NIHR, with the aim of informing the development of policies and strategies designed to facilitate the retention of experienced GPs in direct patient care. This RAM panel has been informed by other ReGroup workstreams, including a systematic review, survey work and qualitative studies with GPs (see Appendix 1).

Research Questions

The RAM Panel seeks to address two research questions:

Question 1. What are the key policies and strategies that might (i) facilitate the retention of experienced GPs in direct patient care and (ii) support the return of GPs to direct patient care following a career break?

Question 2. How feasible is the implementation of those policies and strategies?

The RAM Panel Process

A RAM Panel is usually made up of between 7 and 15 participants, who are asked to take part in an online rating exercise over two rounds. In this study, all the materials will be provided via online links.

In **Round 1**, you are asked to use your professional judgement to rate the statements on 'appropriateness' of the policies and strategies using the definitions and scale provided below.

Following the analysis of Round 1 ratings, in approximately 4 weeks you will receive a new web link for **Round 2**. For each statement, this will show the appropriateness ratings from the group and your own rating. You will be asked to review your original rating and are given the opportunity of changing in light of the group ratings if you wish to. In addition, where statements are rated as appropriate, you will be asked to provide a 'feasibility' rating. The final analysis will take place after Round 2 and the statements rated, by consensus, as appropriate will be presented alongside the feasibility ratings. The RAM panel results will then be used to inform a wider Stakeholder Consultation which is the final workstream for the ReGROUP project.

Appropriateness rating scale

Extremely inappropriate	1	2	3	4	5	6	7	8	9	Extremely appropriate

A policy or strategy statement is called "**appropriate**" **when the expected benefits exceed the expected risks.**

Here, the **expected benefit** is assumed to occur when, after applying the potential strategy or policy approach, GPs are more likely to continue to provide clinical care without quitting or substantially reducing their commitment. This may occur directly through the use of targeted incentives, or indirectly by increasing an individual's confidence and/or competence, or morale.

The **expected risk** is that the potential strategy or policy approach will have no impact on GPs' intentions to quit or substantially reduce their commitment, and/or that it might have unintended consequences that might exacerbate the problem.

When rating the appropriateness of each policy or strategy statement use your best professional judgement and please do not consider cost implications in making this judgement.

Consideration of benefits and risks should take into account issues relating to access, equity, and the safety of the health care service being provided combined with patient experience. At the very least, the policies and strategies will maintain the current levels of service and patient experience, and at best, it should contribute to improvements.

Feasibility rating scale

Definitely not feasible	1	2	3	4	5	6	7	8	9	Definitely feasible

A policy or strategy statement is called “**feasible**” when:

- it can be implemented either at national, regional, or local level;
- with a reasonable prospect of implementation taking place within a *five year* period; and
- it is deemed of reasonable cost, or judged not to not impose an inappropriate burden on the health-care system.

Section 2: Selecting supporting research and published evidence

Generating the list of statements

The key theme in the literature on factors associated with taking a break, quitting or substantially reducing hours for direct patient care is **GP job satisfaction** (see Appendix 2 for detailed rationale). Evidence has found that GPs with low job satisfaction are less likely to remain in direct patient care and are actively considering options such as relocation, changing careers, or opting for earlier than planned retirement age.

In developing and organising the statements (see Appendix 3 for inclusion and exclusion criteria), we focused on areas linked to GP job satisfaction. This was informed by (i) best available research evidence, (ii) UK policy documents and recommended areas for future development linked to GP retention (as of Jan 17th, 2017), and (iii) evidence derived from ongoing research undertaken as part of the ReGROUP project.

The statements are based on components of policies or strategies that can provide support and incentives to retain GPs in direct patient care (i.e. stop them reaching that situation or change their mind), slow down the pace of exit where it is inevitable, or enable faster and smoother entry/re-entry after a period of absence.

Sources of evidence used to inform RAM panel statements

Evidence from two systematic reviews and two main reports provide the evidence-base for this study:

1. **Why do primary care doctors quit direct patient care? A Systematic Review of the empirical research** (Anderson et al., unpublished ReGROUP Workstream)

2. **A systematic review of strategies to recruit and retain primary care doctors** (Verma et al., 2016)
3. **Recruitment and Retention of the Health Workforce in Europe** (Barriball et al., 2015)
4. **General practitioner recruitment and retention: an evidence synthesis** (Peckham et al., 2016)

Due to the wealth of evidence from reviews, data were not routinely sought from individual studies, with the notable exception of two recent studies testing the development of interventions for retention of GPs in England:

5. **Lost to the NHS: a mixed methods study of why GPS leave practice early in England** (Doran et al., 2016)
6. **General practitioner recruitment and retention** (IPSOS MORI Report 2015)

Detailed evidence from these six reports are presented in **Appendix 2** and some of the illustrative quotations from GPs from the ReGROUP qualitative study are presented in **Appendix 4.**

Section 3: How has current policy informed the RAM panel statements?

We have drawn on the plethora of current/forthcoming policies and strategies being proposed/implemented relating to the GP Five Year Forward View (NHS England, 2016) for England, which builds on some of the jointly agreed actions through the Building the Workforce 10 Point Plan (NHS England, 2015). Most of the workforce-based schemes put forward by NHS England centre on creating or supporting the roll-out of financial incentives for GPs to enter or remain in general practice. To date, the following are being taken forward in England:

1. The Retained Doctor Scheme

2. GP Career Plus

3. NHS GP Health Service

4. National GP Induction and Refresher Scheme

5. Targeted Enhanced Recruitment Scheme

6. Targeted Investment in Recruiting Returning Doctors Scheme

7. Introducing a wider workforce to general practice such as the Clinical Pharmacy role

In addition to the schemes specifically addressing workforce, there is a relevant scheme for workload which is aimed at supporting practices who are at risk of closure.

8. Building general practice resilience

Although these schemes are nationally funded and managed, a variety of different stakeholders are responsible for implementing the schemes, such as local NHS England and Health Education England teams, and in the case of retainer schemes, through the general practice. There are also opportunities to apply for pilot project funding as part of some of the schemes including GP Career Plus, which are expected to be reporting outcomes in the next 6 to 18 months.

A summary of the current government schemes and announcements are presented in **Appendix 5** and more information is available through NHS England.

Other policy and strategy reports have informed statement selection. These included the open letter from the Royal College of GPs on targeting retention schemes for older GPs (RCGP, 2016), the King's Fund and Nuffield Trust (2013) report on new organisational models for primary care, and the GP Taskforce Report (2014). The Five Year Forward View (NHS England, 2014) also included policy directions of relevance to primary care and this was included where relevant.

It is likely that some of the national schemes may cover some of the potential policies and strategies included in the statements as part of this research project.

Finally, in developing the statements for this research project, we took into account the existing national schemes. Some statements may appear to be similar to the existing national schemes, and in such cases, we have included additional or alternative approaches or components that require testing.

New announcements and results from pilot sites are expected as this work moves forward. This information was updated on 17 January 2017.

Appendix 1: ReGROUP Workstreams

Workstream 1: A systematic review of empirical research conducted in the UK describing factors influencing GPs decisions to quit patient care.

Workstream 2: A census survey of all GPs in South West England was undertaken to describe the current and projected GP capacity within the next five years in the region.

Workstream 3: Qualitative interviews were carried out with GPs and other members of the general practice community in South West England, to identify the content of potential policies and strategies that might support the retention of GPs in direct patient care.

Workstream 4: (the current study) A RAM expert panel assessment of the likely appropriateness and feasibility of proposed policies and strategies

Workstream 5: Building on the census survey's assessment of current and projected GP capacity, we are using supply-demand modelling techniques to identify supply-demand imbalances at practice level that are likely within the next five years.

Workstream 6: National stakeholder consultation meetings will be led by the Primary Care team with members of key stakeholder groups who have expertise and involvement in GP workforce planning.

Appendix 2: Research Evidence

The evidence reported in this appendix has been used to develop the policy and strategy statements tested in this RAM study.

1. Why do primary care doctors quit direct patient care? A Systematic Review of the empirical research (ReGROUP Workstream: Anderson et al, in preparation for publication – please do not reproduce or circulate without permission)

Background/aims: This systematic review contributed a conceptual framework and detailed assessment of factors associated with UK GPs' decision to quit direct patient care, take career breaks from general practice, or return to practice after a career break. It also identified the evidence regarding potential components of policies and strategies aimed at retaining experienced GPs in direct patient care.

Evidence extracted: Following a systematic review process, 28 UK studies were identified, including 5 qualitative studies and 23 quantitative studies. There were also a total of 13 non-UK studies (12 quantitative and 1 qualitative) included in the review. Non-UK studies were mainly from Australia, Canada, New Zealand, the Netherlands, and Ireland. The quantitative studies were mostly surveys. The qualitative studies were all semi-structured interviews with practising or retired GPs.

Quality of evidence: Most of the quantitative studies were of good methodological quality in relation to key question items. However, most of the analyses were restricted to presenting the associations between two or three parts of the intervention. For non-UK studies, the generalisability of findings to the UK context was deemed limited due to fundamental differences in the way general practice care is organised and delivered.

Conceptual framework: Importance of GP job satisfaction

This review concluded that job satisfaction is the key indicator of whether a GP will successfully adapt and remain in practice, or will become overwhelmed by external influences and pressures and leave the profession. Many GPs report that job satisfaction directly relates to the quality of the doctor-patient relationship, and is dependent on the time available for GPs to spend with their patients. Figure 1 summarises the key 'pulls' that influence GP job satisfaction.

GPs with high job satisfaction: This group describe feeling supported by good practice relationships. The factors included the cultivation of a particular frame of mind, professional autonomy, good administrative support, flexible working, good adaptation to the current context and the pursuit of other interests.

GPs with poor job satisfaction: This group report feeling overworked and unsupported. Many feel part of an over-bureaucratised system, and describe being at the front-end of a service unable to deliver what it promises. Combined with changing relationships with patients and interfaces with

secondary care, and the gradual sense of loss of control over large parts of the job, many GPs report a reduction in job satisfaction. Lack of time with patients is perceived to compromise the ability to practise patient-centred continuity of care and, with it, compromise GPs' professional autonomy and values, resulting in further diminished job satisfaction. Once job satisfaction has become negatively impacted, the combined pressures of increased patient demand and workload together with other stress factors such as poor IT resources, negative media portrayal, poor practice relationships and a "bullying" or "blame" culture has left many feeling unsupported and vulnerable to burnout and ill health, and, ultimately, to the decision to leave general practice.

Overview of factors influencing GP retention within the workforce

GPs in different countries leave general practice due to a wide range of factors; both negative job-related 'push' factors and positive leisure and home life related 'pull' factors. While some factors clearly operate at an individual, personal level – such as the financial ability to retire, health, family and marital circumstances, or good/poor relationships between practice partners – other factors operate at the level of the whole profession or the local or national health system (e.g. media portrayal of GPs, service reform and performance targets, CQC inspections and professional revalidation).

Key job factors: Four closely related, 'higher level' factors seemed to play a major part in decision-making about both early retirement and part-time working: workload, job (dis)satisfaction, work-related stress and work-life balance. These higher-level factors were prominent in studies of both intention to quit or to reduce work hours and in studies of actual decisions to quit or work part-time. Many other detailed factors have been identified that either underlie the four higher level factors (e.g. health service reform fatigue, or deteriorating or unsupportive practice partner relationships) or may combine to influence an individual GP's decision to quit general practice or reduce hours.

Cultural norms: Both the questionnaire survey and the qualitative interview evidence indicate that it is not just 'unhappy GPs' (e.g. those with poor job satisfaction and high workload) who wish to reduce their hours or retire early. Early retirement is now a cultural norm and lifestyle choice within the medical profession for those who can afford it, and who wish to spend more time pursuing their own interests or caring for their family or other loved ones.

Gender and working: While there were differences between male and female GPs in their intentions or preferences for part-time working, these were inconsistent between studies and did not adjust for current hours worked, so it is difficult to draw clear conclusions. However, overall younger female GPs and older male GPs were generally more likely to want to work part time. Only groups already working reduced hours wished to increase their hours. One survey and the qualitative evidence synthesis suggested an association between opportunities for part-time working and delaying retirement. That is, for some GPs, being able to work part-time (and more flexibly) may incentivise them to retire later.

Figure 1: Summary of the factors influencing GP job satisfaction and retention in the workforce

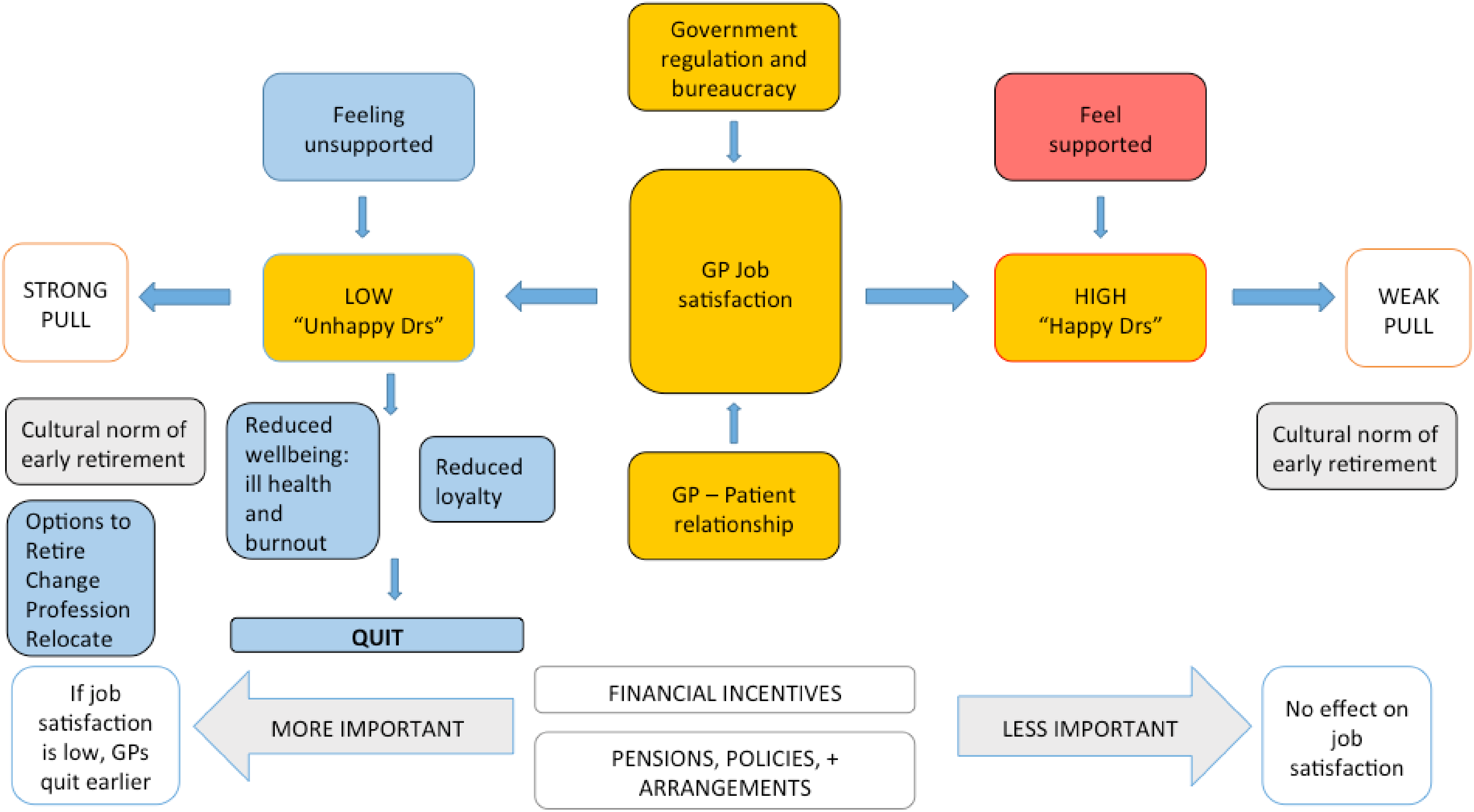

Role of financial incentives: There is no evidence that financial incentives would encourage GPs to remain in the workforce and discourage early retirement, and in fact such incentives may have the opposite impact.

Career breaks: GPs' intentions to take a 'career break' appear to be more influenced by a specific range of 'pull' factors than by negative 'push' factors to do with the job or workload. The main reasons GPs say they will be taking a career break are to work abroad, to have or look after children, or to engage in research or further study. Although the stated reasons for intending to take a career break seem somewhat different to those related to intending to permanently quit patient care, many of the barriers that they say would prevent them from returning to work as a GP relate to negative perceptions that also drive quitting the GP profession (e.g. high workload, low job satisfaction, unsociable hours, excessive administrative work).

Interventions to improve GP retention

There is clearly scope to influence the retention of GPs in the primary care workforce at a number of levels in the health system.

Given the consistency and strength of perceptions about **workload and job-related stress**, either additional staff resources and/or methods of working that enable care to be delivered in a more person-centred way and with less administrative burden would help. Much of the workload related stress appeared to be associated with the higher administrative burden of being a GP or a practice partner. However, some of that stress was also associated with the experience of having to see more patients and/or more complex patients, but with the same traditional constraints on appointment times. Many of these issues can be affected either adversely or for the better by national policies and initiatives. National policies to enable financially stable arrangements when partners wish to retire or to sell their share in the business may also benefit GP retention.

At the practice level, larger practices appear to have more scope to **offer flexible or part-time working**, or simply be a more **supportive working environment**. Such part-time or flexible working in the short-term might enable many GPs to retire later than they otherwise would have. However, the evidence is conflicting, as there was some evidence that very large practices may not sustain such flexibility and support.

A significant minority of GPs also cited **practice partnership relationship problems** as a key factor leading some to quit patient care entirely. So more consistent 'HR-style' workplace health and wellbeing support for GPs, as well as direct support to reduce workload demands and the administration burden may be a part of the solution.

The evidence relating to **potential financial incentives** in the form of additional salary was limited and mixed. On the one hand, perceptions of reducing GP income and dissatisfaction with remuneration are cited in a recent survey study as being a key driver for those quitting

general practice. On the other hand, qualitative interview evidence suggested that increasing pre-retirement salaries was unlikely to help GP retention, and might even increase opportunities to leave practice earlier than planned.

2. Systematic review of strategies to recruit and retain primary care doctors (Verma et al., 2016; online link)

Review aims: Verma et al (2016) carried out a review of interventions specifically for primary care doctors. Fifty-one studies and 42 interventions were identified, mostly from the USA, Canada and rural Australia.

Types of intervention identified: The interventions relating to retention were under nine broad categories of: retainer schemes, re-entry schemes, support for professional development or research, specialised recruiters or case managers, well-being or peer support initiatives, rural or underserved postgraduate training, marketing, delayed partnerships, financial incentives and mixed interventions.

Quality of evidence: It was reported that all the studies were of low methodological quality and only 15 of the 51 studies included studies with a comparison group and these all related to the recruitment schemes with weak evidence that postgraduate placements in underserved areas improved recruitment. Conclusions could not be drawn for majority of the intervention categories.

Key findings: The generalisability of the evidence base to the UK setting is limited due to differences in the delivery off primary care between nations. When drawing comparisons with the existing UK recommendations, the authors concluded that the “research did not find sufficient evidence to support or refute” the funding of a returners scheme as recommended in the UK GP taskforce report. The systematic review also recommended the need to develop a stronger evidence base for the solutions aimed at maintaining the primary care doctor workforce.

3. Recruitment and Retention of the Health Workforce in Europe (Barriball et al., 2015; online link)

Aims/method: This report brings together a literature review, recruitment and retention practices and case studies for healthcare professionals as part of a study funded under the EU health programme. The review included primary studies (n = 60) and reviews (n = 11) from the peer reviewed literature as well as grey literature (n = 50) from 27 EU and non-EU countries. A total of 39 interventions (30% of the total and the highest for all the countries) were identified in the UK.

Types of intervention identified: The recruitment and retention interventions were based on the WHO's (2010) classifications including education (e.g. time for CPD, mechanisms for career progression), regulation (e.g. revisions of scopes of practice, employment contracts or in remuneration mechanisms), financial incentives (e.g. salary incentives, training opportunities), professional and personal support (e.g. flexible working hours, exemption from night and weekend shifts), mix/other types of interventions (e.g. combining education with professional support)

Key Findings: Based on the conclusions drawn in this report, single interventions have limited effects over time. Bärnighausen, and Bloom (2009) and Misfeldt et al (2014) reviewed the literature on the topic and came to similar conclusions: higher wages appear to have a positive influence on job satisfaction initially. However, there is evidence that the effectiveness of financial incentives on retention declines after five years, compared to other factors such as a positive work environment.

Examples of approaches used in different parts of Europe were discussed in the report. As well as highlighting the role of grants and financial incentives, return to practice schemes for healthcare professionals usually involved education measures with attractive financial conditions and the allocation of mentors. Professional and personal support were seen as important facilitators for recruitment and retention especially during transition periods, measures to improve the quality of the work environment, and planned reductions in end of career working time without salary penalties and bonus for those who were working full-time (in Belgium between 2005 and 2010).

There was a strong theme on health and wellbeing for the workforce and this could be related to labour laws and conditions for specific groups with some examples as follows:

a. Dr DOC programme in South Australia: health and well-being programme for rural GPs included funding a GP provide the full spectrum of health care including an emergency support line, health check-ups, and mental health support for their colleagues. The programme evaluation shows significant improvement in the level of feeling of social support and decreases in GPs feeling unsupported, in crisis with no help, and GPs feeling that their mental health is suffering.
b. Nottingham University programme in the UK: a Health and Wellbeing Coordinator position was recruited to implement out programmed activities delivered in partnership with external organisations, e.g. Sustrans, and Occupational Health Nurses. Sickness monitoring data is included as part of evaluation.
c. PAIME in Spain: a programme for the promotion and protection of physicians' health predominantly for over 51 years. They also offered various clinical programmes and assistance for those with mental health problems, and additions.
d. Sozial-Holding der Stadt Monchegladbach: a health and social care organisation in Germany put into place health and age management policies providing further education/CPD opportunities, flexible working arrangements, positive images of ageing in the company, and free mental health support days for all its employees.

A key message was that making incremental changes allows for subtle adjustments which can then tested, and that the stage in their career is also important. Thus the goal is to offer "interventions with enough freedom to allow different actors to select the elements that suit their needs and skills set, but with sufficient structure to ensure that all actors work towards a common goal" (p.42).

Details of the interventions identified in the literature review can be found by clicking here.

3. General practitioner recruitment and retention: an evidence synthesis (Peckham et al., 2016; online link)

Aim/methods: An evidence synthesis of published reviews and primary studies relevant to the UK context was also undertaken as part of the wider study to support the review of the 10 Point Plan commissioned by NHS England and Department of Health. The topics specifically included identifying the efficacy of marketing campaigns, examining the impact of training programmes, financial incentive schemes, non-financial incentives, and changes in workforce structures and their impact on recruitment and retention. One of the objectives of the 10 Point Plan – supporting those who wish to return to general practice was not included as part of this review and therefore it is not possible to draw conclusions for this objective from this review. This is because relevant papers were not identified in the initial analysis of the reviews. Table 1 provides a summary of the types of interventions considered and the conclusions drawn from this report.

Table 1. Factors highlighted in the literature to support development of specific policies and strategies

10 Point Plan	Evidence in GP literature*	Factors highlighted in the literature to support development of specific policies and strategies
5. Investment in retainer schemes	No clear evidence	Widening the scope of remuneration and contract conditioner:<br>- Reduce the income differential between general practice and hospital work<br>- Remove the disincentives for less than full-time employment, widening of the employment mechanisms open to GPs such as authority-organised salaried schemes
6. Improving the training capacity in general practice	No clear evidence	Sub-Specialisation and portfolio careers where doctors might gain skills in arrange of specialities and practices – some or all of the at any one time.

10 Point Plan	Evidence in GP literature	Factors highlighted in the literature to support development of specific policies and strategies
7. Incentives to remain in practice	No clear evidence	
8. New ways of working	No clear evidence	Varying time commitment across the working day and week:<br>- Part-time, job share; temporary and short-time available, whatever a GP's employment status and career stage.<br>Offering a wider choice of long-term career paths:<br>- Locum and associate positions equal to full-time principal posts<br>- Activities such as research and training in management skills<br>- A part-time educational post, or hospital attachment<br>- Job mobility as a way to progress (a more positive vision of mobility).
Other	Evidence	Factors that increased satisfaction:<br>- Job autonomy / diversity /variety<br>- Social support, relationship and collaboration with colleagues/patients<br>- Academic hospital and centres / teaching medical students and advanced students<br><br>Factors that decreased satisfaction:<br>- Too many working hours, low income / compensation / workload / not enough time / high demands / lot of paperwork / little free time<br>- Lack of support / lack of colleagues<br>- Lack of recognition<br>- Bureaucracy / practice administration

Key findings: The limitations of the published evidence in supporting policies and strategies were highlighted and it was reported that most of these focussed on recruitment of GPs into underserved areas. Although discussed more in relation to recruitment, promoting general practice (through marketing strategies) did not have a clear evidence base in the literature, although it was concluded that having role models and "enhancing the status, contribution, career advancement and rewards of primary practitioners" were viewed as facilitative factors.

Consistent with the findings of Anderson et al, **increased job satisfaction** was seen as the key driver for retention. These include increasing supportive structures that facilitate job

autonomy, social support, relationship and collaboration with colleagues/patients, and linking to academic centres with an association to high quality education establishments.

Organisational and bureaucratic barriers added to dissatisfaction and based on one of the qualitative studies, the discussion of financial incentives such as holding back a lump sum ('golden handcuffs') to avoid early retirement was described as impacting negatively on their professional integrity.

4. Lost to the NHS: a mixed methods study of why GPS leave practice early in England (Doran et al., 2016; online link)

Aims/method: Doran et al (2016) conducted, a high quality survey of GPs who had left direct patient care in England during a five year period (from 2009/10 to 2014/15). Doran and colleagues asked detailed questions to elicit their reasons for leaving general practice, plus some questions about attitudes to 'Retainer' schemes and the GP 'Induction and Refresher' scheme. Some of the findings were presented as interim results (Doran et al, 2014; 2015).

Key results: The main categories of reason for leaving direct patient care, given by the 142 GPs who responded, are shown in Figure 2, with the frequency of more specific reasons by GP leavers presented in Figure 3. Although 'personal factors' were the most common category reported, followed by pressure of work, the two were closely related i.e. 'personal factors' were related to workload and job satisfaction (feeling overworked or burnt out, poor work-life balance, and no longer enjoying being a GP). Similarly, working hours being "too long", or 'incompatible with other things that were important to me' were cited as reasons by almost a half (47%) and over a third of GP leavers (35%) respectively. For some, workload pressures appeared to be compounded by perceived loss of autonomy and professional control (44%), high patient expectations (34%), and the perception that the 'workload required for my yearly NHS appraisal was too high' (29%). Financial factors encompassed a perceived imbalance between remuneration and the amount of work (34%), falling income (34%), but also for a significant minority (21%) who felt that their NHS pension was under threat.

Practice-level factors that were reported to be important included having a non-clinical workload that was too high (50%), feeling isolated and lacking professional support (22%), and having colleagues that they no longer felt able to work with (13%). Policy or national-level influences included not liking the media's attacks on the medical professions (57%), the 'goalposts being moved too often' (56%), not liking the target-driven approach to patient care (52%), and being unhappy with changes to the role of the GP (44%).

Figure 2. Main categories of GPs' stated reasons for leaving direct patient care (2014)

*Source: Doran at el (2015). *'Personal factors' included a number of workload-related factors such as: feeling overworked or burnt out, poor work-life balance, and no longer enjoying being a GP.*

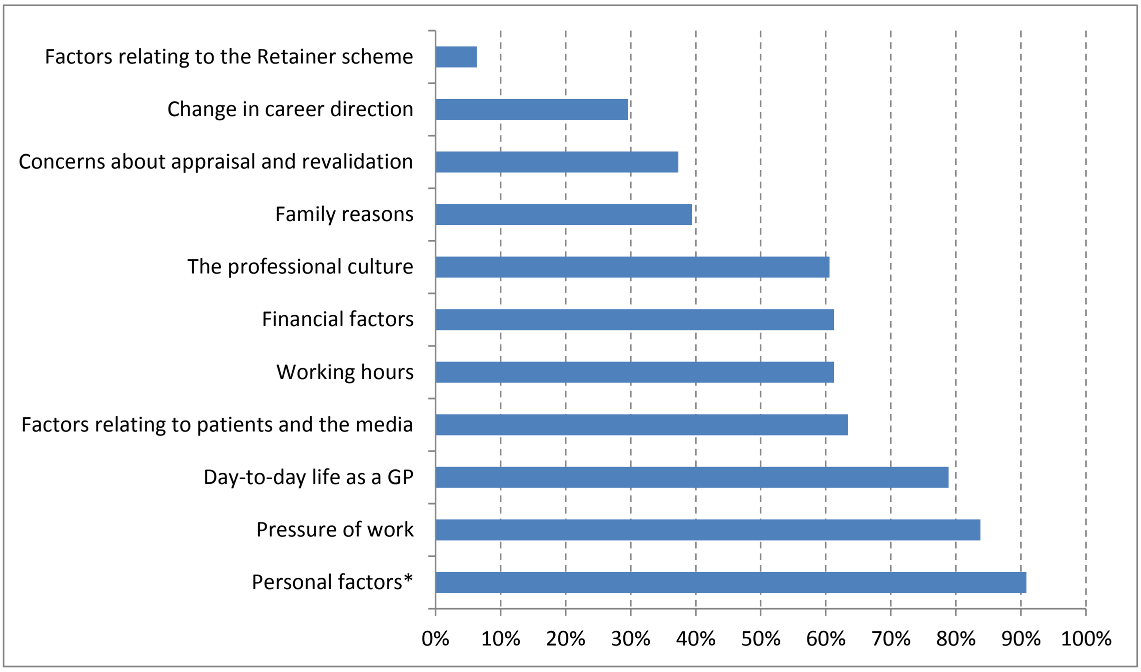

Figure 3. GPs' most common specific reasons for leaving direct patient care

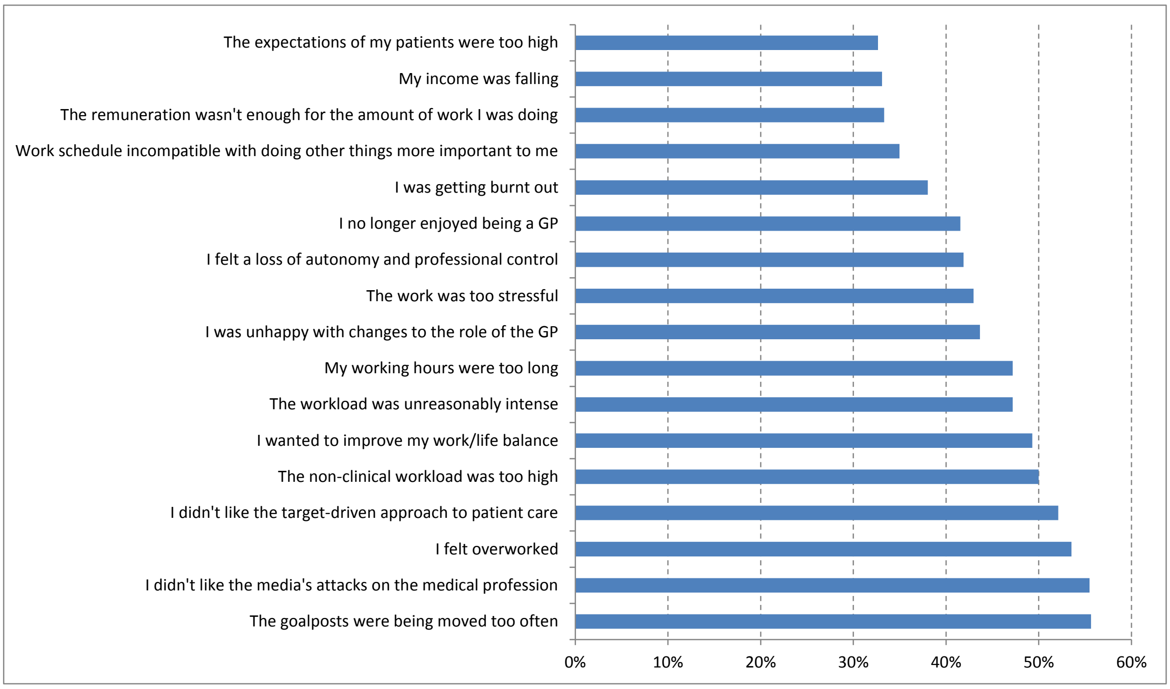

Source: Doran et al (2015).

5. General practitioner recruitment and retention: an IPSOS MORI Report (2015; online link)

Aim/method: A qualitative programme of research was undertaken as part of the wider study to support the review of the 10 Point Plan as commissioned by NHS England and Department of Health. This included 41 in-depth interviews with GPs with serious intent of leaving general practice and facing personal challenges to remaining, and 23 in-depth interviews with GPs who had left or were returning back to general practice. The *"Looking to the Future: the Recruitment, Retention and Return of GPs Report"* identified factors such as the satisfying aspects of the role and the 'emotional desire to want to be a GP' was being affected negatively by 'corroboration of social, cultural, organisational, and system-based factors'. Being able to maintain the emotional connection, self care and reducing isolation in the workplace were all identified as part of the solution.

Types of intervention tested: Incentives were tested as part of the interviews and these fell into three types:

- **One-off investments in GPs** included bursaries, vouchers etc. and participants raised questions about creating a situation where the Partner would be absent from Practice and therefore creating new workload for those who were remaining in the practice.
- **Incentives to provide a 'nudge'** such as rotations into hard to reach areas and relocation grants were tested. These were viewed as insufficient to address the retention issue and were more suited to the GPs early on in their career who were willing to consider filling the gap.
- **Incentives based around the development of schemes or ideas already in place** including portfolio careers and retainer schemes were discussed. Mixed views were encountered. For example, although there was a strong appeal for portfolio career schemes, it was reported that many GPs are able to organise these themselves and that the retainer schemes still does not address the fundamental issues around child care responsibilities, working hours etc.
- **Removal of Practice level disincentives** were considered to be important levers for retention. Five long term strategies were identified: (1) getting GPs on side and feeling valued, (2) tackling professional identity with guidance on GPs roles and responsibilities, and managing expectations (3) ways of working to build resilient systems with streamlining, and tackling lack of flexibility, (4) service design and viable models of family practice for both size and clinical responsibilities, and (5) future proofing retention strategies with time testing and a plan for sustainability.

Appendix 3: Statement inclusion and exclusion criteria

Inclusion criteria
1. The policies and strategies have been extracted electronically or extrapolated from key sources and therefore represent areas have already been identified by research, national policy or equivalent publications.<br>2. The policies and strategies are seeking to address known barriers and facilitators to increasing retention, reducing intention to leave, or encouraging re-entry into direct patient care (as identified from the key sources and internal contributions from the ReGROUP workstreams).<br>3. The policies and strategies seek to build on known and existing schemes or approaches in order to increase the potential impact on increasing retention, reducing intention to leave, or encouraging re-entry into direct patient care. The interventions proposed or tested may also be within the context of increasing job satisfaction. In these cases, the interventions is at best be a surrogate outcome for improving GP retention.
**Exclusion criteria**
1. The policies and strategies do not fit the UK general practice context in terms of how GPs, practices and commissioning is managed.<br>2. It is known that it will take more than 5 years to action the relevant policies and strategies.<br>3. Policies and strategies which are not described in current research and policy documents. The latter includes innovations that might plausibly used to facilitate GP retention, but which are currently untested or not specified with the literature.

Appendix 4: Illustrating the context with quotations from GPs

The qualitative research study (analysis in progress) carried out as part of the ReGROUP study has released some of the interview quotations as part of illustrating some of the areas that have been highlighted through the research evidence.

Support is needed at all levels:

"Our practice did have a supportive culture and so most of the young doctors who come and do work in our practice would learn that this was an environment in which they could work…we were able to make our practice attractive, not by being massively high earning, but actually by offering a supportive environment in which to work." GP104 - early retiree

"I think the government and the GMC are particularly culpable in eroding that sense of belonging or protection. Not protection, that's the wrong (word), it sounds like we'll be protected if we do things wrong…but just that kind of buffering system to be able to deal with what goes on in the daily GP life." GP505 - staying GP

Fear of (safe) practice being compromised impacts upon GP decision making:

"So for me, it felt like it wasn't safe practice, seeing such a high volume of patients and being pulled in so many different directions." GP412 - early leaver

"this isn't about always saving money and making money go further, this is about making the workforce sustainable. And actually if you make it safer for doctors then you make it safer for patients, you get a better quality service, you get a service that saves money" GP410 - intending to take a career break

Accumulative, compounded, and combined factors lead to the decision to leave:

"It's really like an insidious, drip drip drip thing really that's been happening for 10 plus years, really. There's more and more and more things coming our way." GP207 - intending early retirement

"I've just become more and more desperate, actually … in past years I have just felt terribly angry with the way things are going and now I think, "I can't actually do anything more about it." And if I could do anything but vote with my feet, but ultimately it's the only vote which they're going to listen to. And one does definitely feel guilty about leaving one's partners trying, effectively trying to well, not pick up the pieces, but keep the boat afloat as it were. But, I can't ...there just comes a point, you've got one life and one can't sacrifice oneself totally, so, yeh, I'm going to go" GP208 - intending early retirement

Appendix 5: Summary of the current government schemes

All of the schemes below are currently being implemented, and to the best of our knowledge, there are no formal evaluations taking place.

1. The Retained Doctor Scheme (online link)

This scheme provides financial incentives and development support for GPs working between one and four sessions per week. There are session-based payments (at higher rate than previous years) for general practices working with retained GPs and financial contributions towards professional expenses directly aimed at GPs.

These are available for up to 36 months until 30 June 2019 and implementation is managed by general practices.

2. GP Career Plus (online link)

This is a pilot project aimed at 80 GPs at risk of leaving the profession across 10 areas. The details for the scheme are not available as it will be dependent on the models put forward by the areas. However, the policies and strategies put forward are expected to provide flexibility and support for those who continue in a clinical capacity covering vacancies, sick leave etc., carry out specific types of work such as home visits, or provide leadership through clinical training, mentoring, and other support for practices in crisis or in under-doctored areas.

This is a 12 month pilot managed by NHS England and is taking place in 2017/18.

3. NHS GP Health Service (online link)

The Hurley Clinic Partnership will be providing access to mental health support for general practitioners and trainee GPs who may be suffering from mental ill-health including stress, depression, addiction and burnout. This is available to GPs and GP Trainees who are registered on the National Performers List in England as well as to those who are looking to return to practice after a period of absence. This is a self-referral scheme with a dedicated helpline, online access (email, website, and smartphone app), and consultations being made available through the 13 NHS England local team areas.

This scheme is funded by NHS England for five years launching in January 2017.

4. National GP Induction and Refresher (I&R) Scheme (online link)

This is aimed at GPs who have taken a career break including spending time working abroad, as well as introduction of overseas GPs who have qualified outside the UK. There are three components including (1) financial support through bursaries, and assistance with professional fees including removal of assessment fees, (2) increased practical support through a national support team and dedicated account manager, and (3) easier return to practice including reduction in length of placements based on scores in the I&R assessments. The Portfolio route is linked to this scheme for those who are working as a GP overseas and return to NHS within 5 years. Through this route, return is facilitated with a short period of refresher training as long as the portfolio submission is successful.

Additional funding has been allocated by NHS England in April 2017 for this scheme and are higher proportion of the GPs on the I&R scheme are working in the London area.

5. Targeted Enhanced Recruitment Scheme (online link)

This is specifically for GP Trainees committed to working in areas based on the number of training places with the lowest fill rates between 2013 and 2016. Somerset and North Devon have been identified in the South West. A one-off payment of £20,000 is offered as a financial incentive to train for three years in these areas. Although this is not directly relevant to the current research project, it provides an indication of current policies in place for the GP workforce.

The agreement is between the trainee and HEE and the pilot funding for 2016 has been extended to 2017 for 144 places.

6. Targeted Investment in Recruiting Returning Doctors Scheme (online link)

This is aimed at general practices to support recruitment in practices where they have historically encountered recruitment issues (with vacancies held for a minimum of 12 months). The pilot scheme is underway with relocation allowances, education bursary, and locum cover for the GP undertaking educational sessions. These strategies are expected to increase the attractiveness of these posts for new recruitment and fifty practices are participating in the pilot scheme.

The scheme is managed by the NHS England local teams and Commissioning Support Units are supporting the general practices in promoting vacancies to improve recruitment.

7. Introducing a wider workforce to general practice such as the Clinical Pharmacists (online link)

Following on from a pilot to support the expansion of clinical pharmacy, new funding has been released to train and develop more clinical pharmacists. General practices have had the opportunity to apply for the funding and if selected will receive funding for 3 years to recruit and sustain the roles.

The project is managed by NHS England in collaboration with Health Education England, the Royal College of General Practitioners and the British Medical Association's GP Committee are working with the Royal Pharmaceutical Society.

8. Building general practice resilience (online link)

This is aimed at practices at risk of closure and provides support including specialist advice, coaching, mentorship, and rapid intervention and management support. The practices are self-determined and the aim is to support workforce and HR issues including management, recruitment and capacity planning.

The scheme is managed and delivered by NHS England local teams and in place for 4 years up to 2020.

References

Bärnighausen, T., & Bloom, D. E. Financial incentives for return of service in underserved areas: a systematic review. *BMC Health Services Research*, 9:86. 2009.

Barriball L, Bremner J, Buchan J, Craveiro I, Dieleman M, Dix O, Dussault G, Jansen C, Kroezen M, Rafferty AM, Sermeus W., *Recruitment and Retention of the Health Workforce in Europe*. European Commission, 2015.

Brook RH, Chassin MR, Fink A, Solomon DH, Kosecoff J, Park R. *A Method for the Detailed Assessment of the Appropriateness of Medical Technologies*. Santa Monica: RAND, 1991.

Doran N, Fox F, Taylor G, Harris M. E*arly GP leavers interim report: report to HEE & NHS England dated 4th May 2014*. Bath: University of Bath, 2014. http://opus.bath.ac.uk/47271/ (accessed Jan 2017).

Doran, N., Fox, F., Rodham, K., Taylor, G. and Harris, M. (2016) Lost to the NHS: a mixed methods study of why GPs leave practice early in England, *British Journal of General Practice*, 66(643), pp. e128–e135.

Fitch K, Bernstein SJ, Aguilar MD, Burnard B, LaCalle JR, Lazaro P, et al. *The Rand/UCLA Appropriateness Method User's Manual*. Santa Monica: RAND, 2001.

GP Taskforce, *Securing the Future GP Workforce - Delivering the Mandate on GP Expansion*, 2014. Available from https://www.hee.nhs.uk/sites/default/files/documents/GP-Taskforce-report.pdf (last accessed Jan 2017).

IPSOS MORI Report, *Looking to the future: the recruitment, retention and return of GPs*, 2015. Available from https://www.england.nhs.uk/commissioning/wp-content/uploads/sites/12/2015/01/gp-retention-return-imp-summary-rep.pdf (last accessed Jan 2017).

King's Fund and Nuffield Trust. *Securing the Future of General Practice: New Models of Primary Care*, 2013. Available from: http://www.nuffieldtrust.org.uk/publications/securing-future-general-practice (last accessed Jan 2017).

Misfeldt, R., Linder, J., Lait, J., Hepp, S., Armitage, G., Jackson, K., & Suter, E. Incentives for improving human resource outcomes in health care: overview of reviews. *Journal of Health Services Research & Policy*, 19(1), 52–61. 2014.

NHS England, *Five Year Forward View*, 2014.

NHS England, *Building the Workforce – the New Deal for General Practice*, 2015.

NHS England, *GP Five Year Forward View*, 2016.

Peckham, S, Marchand, C, Peckham, *A. General practitioner recruitment and retention: An evidence synthesis* PRUComm, 2016.

RCGP, *Retention of older GPs*, 2016. Available from http://www.rcgp.org.uk/news/2016/september/retaining-gps-must-be-as-big-a-priority-as-recruiting-new-ones.aspx (last accessed Jan 2017).

Verma, P, John A. Ford, J.A., Stuart, A, Howe, A, Everington, S, Steel, N A systematic review of strategies to recruit and retain primary care doctors, *BMC Health Services Research*, 16: 126, 2016.
