# Supplementary material for: Identifying policies and strategies for general practitioner retention in direct patient care in the United Kingdom: a RAND/UCLA appropriateness method panel study
Source: BMC Fam Pract. 2019 Sep 12;20:130. doi: 10.1186/s12875-019-1020-x (PMC6743144; doi:10.1186/s12875-019-1020-x)
Supplement: Supplementary file 2 — RAM questionnaire. (PDF 419 kb) [file 12875_2019_1020_MOESM2_ESM.pdf]

Assessing the appropriateness and feasibility of policies and strategies for retaining GPs in direct patient care: RAM Statements Round 1

Thank you for participating as a ReGROUP RAM Panel member. The ReGROUP study aims to explore GPs' decision-making about taking a break from or leaving direct patient care, the impact on general practice organisation and management, and possible ways to facilitate GP retention. Please click here for a copy of the information sheet.

In this round, you are asked to use your professional judgement to rate the statements on 'appropriateness' of the policies and strategies. Please use the Evidence Summary for further details on the development of the statements.

To complete this questionnaire online, please go to
http://www.surveygizmo.com/s3/3331169/ram-panel-statements

Please complete your name and email address

Name: ___________________________________

Email: ____________________________________

We are available for telephone and email support, and can also record responses to this questionnaire by phone. Please do contact us for more information or if you have any questions.

Contact details for researcher: Dr Rupa Chilvers: 07908 766971 or r.chilvers@exeter.ac.uk
University of Exeter Medical School, St Luke's Campus, Smeall Building, Magdalen Road, Exeter, EX1 2LU

Sections in the questionnaire

The proposed policy and strategy statements are presented for application at three levels and there are sub-groups identified for some of the statements.

Regional or national level

1) Supporting areas based on risk status
2) Encouraging growth of new GP practices and systems
3) Marketing-based interventions

GP Practice level

4) Focussing on GP Returners
5) Flexible working and managed exits
6) Human Resources Management for GPs

Sub-groups for Section (6) only:

(a) All practices/ Employers
(b) GP practices/ Employers operating in traditionally "hard to recruit" settings

GP level

7) Health and Wellbeing
8) Professional Support
9) Support for portfolio working
10) Employment, contracts and transition
11) Additional support

Sub-groups for Sections 7 to 10:

(a) for all GPs
(b) for GPs who are reaching retirement age and could take their pensions upon exit
(c) for GPs who are not reaching retirement age and cannot take their pension upon exit

Section 11 is only applied to one sub-group:

GPs who are reaching retirement age and could take their pensions upon exit

Appropriateness rating scale (9 points)

Extremely inappropriate	1	2	3	4	5	6	7	8	9	Extremely appropriate

A policy or strategy statement is called "**appropriate**" **when the expected benefits exceed the expected risks.**

Here, the **expected benefit** is assumed to occur when, after applying the potential strategy or policy approach, GPs are more likely to continue to provide clinical care without quitting or substantially reducing their commitment. This may occur directly through the use of targeted incentives, or indirectly by increasing an individual's confidence and/or competence, or morale.
The **expected risk** is that the potential strategy or policy approach will have no impact on GPs' intentions to quit or substantially reduce their commitment, and/or that it might have unintended consequences that might exacerbate the problem.

When rating the appropriateness of each policy or strategy statement use your best professional judgement and please do not consider cost implications in making this judgement.

Consideration of benefits and risks should take into account issues relating to access, equity, and the safety of the health care service being provided combined with patient experience. At the very least, the policies and strategies will maintain the current levels of service and patient experience, and at best, it should contribute to improvements.

Statements for regional or national level (please circle your rating: 1 - extremely inappropriate | 5 - neutral | 9 - extremely appropriate)

Statement	Appropriateness Scale
**1) Supporting areas based on risk status**	
The 'at-risk' status of a Practice or an area can be based on known, anticipated and/or reported factors which may result in a substantial reduction in the availability of their GPs providing direct patient care in line with the health care needs of their local population.	
1.1. In order to assess 'at-risk' status in a commissioning/planning area and taking into account confidentiality:	
(a) GPs should be required to provide 'intention to quit' information regularly to assess areas 'at-risk'.	1 2 3 4 5 6 7 8 9
(b) GPs should be required to complete job satisfaction surveys (or equivalents) regularly to assess areas 'at-risk'.	1 2 3 4 5 6 7 8 9
(c) GP practices should be required to register their organisation's at-risk status.	1 2 3 4 5 6 7 8 9
(d) GP practices should be able to self-register their organisation's 'at-risk' status.	1 2 3 4 5 6 7 8 9
(e) there should be regular audits to identify GP practices 'at-risk'.	1 2 3 4 5 6 7 8 9
1.2. GP practices identified as being 'at-risk' should be:	
(a) targeted with additional support and incentives.	1 2 3 4 5 6 7 8 9
(b) provided with a toolkit to manage recruitment and retention.	1 2 3 4 5 6 7 8 9
(c) prioritised for new/innovative national schemes to support GP retention and/or return to work.	1 2 3 4 5 6 7 8 9
(d/e) managed with an appropriate and sensitive supportive arrangement and this should be	
(d) compulsory	1 2 3 4 5 6 7 8 9
(e) optional	1 2 3 4 5 6 7 8 9
(f/g) allocated a specialist team for managing recruitment and retention, and this should be	
(f) compulsory	1 2 3 4 5 6 7 8 9
(g) optional	1 2 3 4 5 6 7 8 9

Statements for regional or national level (please circle your rating: 1 - extremely inappropriate | 5 - neutral | 9 - extremely appropriate)

Statement	Appropriateness Scale
**2) Encouraging growth of new GP practices and systems**	
(a) New incentive and support packages should be available to GPs and other organisations setting up new practices or new ways of working in under-doctored areas.	1 2 3 4 5 6 7 8 9
(b) New arrangements should be developed so that GPs can become more involved in GP practice management without being partners.	1 2 3 4 5 6 7 8 9
(c) New business models should be developed for GPs who wish to provide care within the NHS but prefer not to own a GP practice.	1 2 3 4 5 6 7 8 9
(d) There should be incentive and support packages for not-for-profit organisations employing GPs to work across GP practices.	1 2 3 4 5 6 7 8 9
(e/f) Hospitals should be permitted to open:	
(e) GP practices with registered lists.	1 2 3 4 5 6 7 8 9
(f) GP practices with registered lists in traditionally "hard to recruit" settings.	1 2 3 4 5 6 7 8 9
**3) Marketing-based interventions**	
(a) There should be a publicity campaign highlighting the experiences of GPs who have successfully been retained in direct patient care as part of a marketing-based intervention aimed at GPs.	1 2 3 4 5 6 7 8 9
(b) The positive experiences of GPs who are providing direct patient care should be consistently shared in a number of ways such as blogs and articles as part of a marketing-based intervention aimed at GPs.	1 2 3 4 5 6 7 8 9
(c) There should be a publicity campaign focussing on managing expectations of patients in line with the resources and constraints of GP based primary care services.	1 2 3 4 5 6 7 8 9

Statements for GP Practice level (please circle your rating: 1 - extremely inappropriate | 5 - neutral | 9 - extremely appropriate)

Statement	Appropriateness Scale
**4) Focussing on GP Returners**	
4.1. GPs who are returning to work after a period of absence or after a career break should have access to 'Health and Wellbeing programmes' to help them manage their re-entry into the workforce. Engagement with such a course should be:	
(a) *compulsory* for such GPs	1 2 3 4 5 6 7 8 9
(b) *optional* for such GPs	1 2 3 4 5 6 7 8 9
4.2. GPs who are returning to work after a period of absence or after a career break should have access to schemes that:	
(a) have a range of routes and options that can be combined in a personal package for re-entry	1 2 3 4 5 6 7 8 9
(b) use a mix of online education and face-to-face meetings to ensure timely access to induction and refresher courses	1 2 3 4 5 6 7 8 9
**5) Flexible working and managed exits**	
(a) GP practices should have systems in place to accommodate flexible ways of working.	1 2 3 4 5 6 7 8 9
(b) GP practices should be able to demonstrate commitment to flexible ways of working through written HR policies, guidelines or equivalents.	1 2 3 4 5 6 7 8 9
(c) HR management support should be available to GP Practices who are actively supporting GPs in combining other career interests with direct patient care.	1 2 3 4 5 6 7 8 9
(d) GP practices should receive guidance on recommended approaches to supporting the staged exit of GPs who are looking to leave direct patient care.	1 2 3 4 5 6 7 8 9
(e) GP practices should receive a toolkit on recommended approaches to supporting the staged exit of GPs who are looking to leave direct patient care.	1 2 3 4 5 6 7 8 9
(f) GP practices should implement strategically planned exits for retiring GPs.	1 2 3 4 5 6 7 8 9

Statements for GP Practice level (please circle your rating: 1 - extremely inappropriate | 5 - neutral | 9 - extremely appropriate)

Statement	Appropriateness Scale<br>(a) All practices/ Employers	(b) GP practices/ Employers operating in traditionally “hard to recruit” settings
**6) Human Resources Management for GPs**<br>Human resources responsibilities should be carried out externally to the employer/practice with responsibility for:		
6.1. ongoing monitoring of how many GPs within an area have requested and successfully implemented flexible working arrangements.	1 2 3 4 5 6 7 8 9	1 2 3 4 5 6 7 8 9
6.2. managing flexible working arrangements for GPs.	1 2 3 4 5 6 7 8 9	1 2 3 4 5 6 7 8 9
6.3. all activities associated with retention of GPs .	1 2 3 4 5 6 7 8 9	1 2 3 4 5 6 7 8 9
6.4. all activities associated with professional development and training.	1 2 3 4 5 6 7 8 9	1 2 3 4 5 6 7 8 9
6.5. implementing standards for working hours and conditions.	1 2 3 4 5 6 7 8 9	1 2 3 4 5 6 7 8 9

Statements for GP level **(please circle your rating: 1 - extremely inappropriate | 5 - neutral | 9 - extremely appropriate)**

Statement	(a) for all GPs	(b) for GPs who are reaching retirement age and could take their pensions upon exit	(c) for GPs who are not reaching retirement age and cannot take their pension upon exit
	Appropriateness Scale		
**7) Health and Wellbeing**			
7.1. Peer support initiatives should be made available to GPs aimed specifically at health and well-being.	1 2 3 4 5 6 7 8 9	1 2 3 4 5 6 7 8 9	1 2 3 4 5 6 7 8 9
7.2./7.3. GPs should have access to their own specialised health care service to ensure a quick and confidential:			
7.2. occupational health care service.	1 2 3 4 5 6 7 8 9	1 2 3 4 5 6 7 8 9	1 2 3 4 5 6 7 8 9
7.3. general health service.	1 2 3 4 5 6 7 8 9	1 2 3 4 5 6 7 8 9	1 2 3 4 5 6 7 8 9
**8) Professional Support**			
8.1. A structured programme of training and support should be made available to all GPs in their first 5 years following qualification as an independent GP to help them establish healthy, productive careers. Engagement with such a course should be:			
(a) compulsory for such GPs	1 2 3 4 5 6 7 8 9		
(b) optional for such GPs	1 2 3 4 5 6 7 8 9		
8.2. GPs should receive business management training and opportunities as a component of updating their skillsets.	1 2 3 4 5 6 7 8 9	1 2 3 4 5 6 7 8 9	1 2 3 4 5 6 7 8 9
8.3. Clinical mentorship should be available to GPs as part of a nationally managed scheme.	1 2 3 4 5 6 7 8 9	1 2 3 4 5 6 7 8 9	1 2 3 4 5 6 7 8 9

Statements for GP level (please circle your rating: 1 - extremely inappropriate | 5 - neutral | 9 - extremely appropriate)

Statement	Appropriateness Scale (a) for all GPs	(b) for GPs who are reaching retirement age and could take their pensions upon exit	(c) for GPs who are not reaching retirement age and cannot take their pension upon exit
**9) Support for portfolio working**<br>Portfolio working includes activities such as medical/NHS management, medical education, research/academia, charitable medical work, commissioning, private sector, clinical informatics, and following additional training routes.			
9.1/9.2. GPs should consider portfolio working as part of their career pathway and this should be			
(9.1) compulsory	1 2 3 4 5 6 7 8 9	1 2 3 4 5 6 7 8 9	1 2 3 4 5 6 7 8 9
(9.2) optional	1 2 3 4 5 6 7 8 9	1 2 3 4 5 6 7 8 9	1 2 3 4 5 6 7 8 9
9.3. Career support should be available to GPs to enable portfolio opportunities to be identified and taken up in a strategic way to inform their future ambitions.	1 2 3 4 5 6 7 8 9	1 2 3 4 5 6 7 8 9	1 2 3 4 5 6 7 8 9
9.4./9.5. Incentives and support packages should be available for those GPs developing portfolio careers who are:			
(9.4) making a substantial contribution to direct patient care.	1 2 3 4 5 6 7 8 9	1 2 3 4 5 6 7 8 9	1 2 3 4 5 6 7 8 9
(9.5) linking their portfolio activities to specialisms/areas that are directly beneficial to local clinical priorities.	1 2 3 4 5 6 7 8 9	1 2 3 4 5 6 7 8 9	1 2 3 4 5 6 7 8 9

Statements for GP level (please circle your rating: 1 - extremely inappropriate | 5 - neutral | 9 - extremely appropriate)

Statement	Appropriateness Scale (a) for all GPs	(b) for GPs who are reaching retirement age and could take their pensions upon exit	(c) for GPs who are not reaching retirement age and cannot take their pension upon exit
**10) Employment, contracts and transition**			
10.1. Where a strong case can be made that there is a financial risk directly relating to the work of the Practice (e.g. ownership of premises), GPs should have access to schemes to reduce financial burden (e.g. buy back schemes for premises).	1 2 3 4 5 6 7 8 9	1 2 3 4 5 6 7 8 9	1 2 3 4 5 6 7 8 9
10.2. GPs should be expected to include regular supervision/mentoring sessions as part of their normal professional activity.	1 2 3 4 5 6 7 8 9	1 2 3 4 5 6 7 8 9	1 2 3 4 5 6 7 8 9
10.3. There should be an agreed maximum in the number of consultations that a GP should be allowed to conduct in a working day in order to protect patient safety as well as the health of the GP.	1 2 3 4 5 6 7 8 9	1 2 3 4 5 6 7 8 9	1 2 3 4 5 6 7 8 9
10.4. There should be contractual changes to encourage longer consultations where appropriate.	1 2 3 4 5 6 7 8 9	1 2 3 4 5 6 7 8 9	1 2 3 4 5 6 7 8 9

Statements for GP level (please circle your rating: 1 - extremely inappropriate | 5 - neutral | 9 - extremely appropriate)

Statement	Appropriateness Scale (a) for all GPs	(b) for GPs who are reaching retirement age and could take their pensions upon exit	(c) for GPs who are not reaching retirement age and cannot take their pension upon exit
10.5. The working hours of GPs should routinely include fully-funded, dedicated time to accommodate the full range of roles (administrative, clinical, training, management, CPD, business) undertaken as part of care professional activity.	1 2 3 4 5 6 7 8 9	1 2 3 4 5 6 7 8 9	1 2 3 4 5 6 7 8 9
10.6. Contracts based on specified programmed activities should be available to GPs to work across several GP practices and on other health related activities.	1 2 3 4 5 6 7 8 9	1 2 3 4 5 6 7 8 9	1 2 3 4 5 6 7 8 9

Statements for GP level (please circle your rating: 1 - extremely inappropriate | 5 - neutral | 9 - extremely appropriate)

Statement	Appropriateness Scale<br>**For GPs who are reaching retirement age and could take their pensions upon exit.**
**11) Additional support**	
11.1. General statements	
(a) A comprehensive flexible careers scheme should be introduced with a view to supporting annualised hours, part-time working, and/or ad-hoc contributions to direct patient care.	1 2 3 4 5 6 7 8 9
(b) There should be financial incentives for such GPs who have maintained a prolonged/sustained period of direct patient care.	1 2 3 4 5 6 7 8 9
11.2. The annual appraisal and revalidation process for such GPs should be reviewed with a view to streamlining and simplifying the process:	
(a) for all such GPs.	1 2 3 4 5 6 7 8 9
(b) for such GPs who have not encountered any concerns in the previous revalidation/appraisal processes.	1 2 3 4 5 6 7 8 9
(c) for such GPs who would like to work with a specified and limited scope of practice.	1 2 3 4 5 6 7 8 9
11.3. Such GPs should be eligible for and offered support to facilitate direct patient care:	
(a) including additional dedicated administrative support.	1 2 3 4 5 6 7 8 9
(b) including medical assistants and other equivalent roles.	1 2 3 4 5 6 7 8 9
11.4. Where appropriate, planned exits for such GPs should include pairing them in job share schemes:	
(a) with GPs returning to practice.	1 2 3 4 5 6 7 8 9
(b) with newly qualified GPs.	1 2 3 4 5 6 7 8 9

Please provide comments on the statements or rating exercise.

Thank you for taking part in this RAM panel. Your responses are very important to us. We will now analyse the data and prepare for the next round. This will be released in March 2017.

If you have any queries, please do not hesitate to contact us directly:
Researcher, Dr Rupa Chilvers: r.chilvers@exeter.ac.uk (07908 766971)
